# Rapid Screening for Hazardous Substances with Regulatory Differences in Milk Between Countries Using Ultra-High Performance Liquid Chromatography Ion Mobility Quadrupole Time-of-Flight Mass Spectrometry

**DOI:** 10.3390/foods14060967

**Published:** 2025-03-12

**Authors:** Qiaozhen Guo, Jing Zhang, Bing Shao, Jie Yin, Yunjia Yang, Yi Yang

**Affiliations:** 1 Key Laboratory of Diagnostic and Traceability Technologies for Food Poisoning, Beijing Center for Disease Control and Prevention, Beijing 100013, China; 2 School of Public Health and Family Medicine, Capital Medical University, Beijing 100089, China

**Keywords:** regulatory heterogeneity, hazardous substances, screening, UPLC-IM-QTOFMS, milk

## Abstract

Regulatory heterogeneity on the maximum residue levels (MRLs) of hazardous substances for food is identified as a challenge of trade between countries. To balance the import and export trade of milk, a high-throughput determination method was established for hazardous substances with regulatory differences. In this paper, we investigated 462 hazardous substances with different MRLs for food based on different countries’ regulations, involving pesticides, veterinary drugs, and some toxins. A mass database was established for these compounds including the basic information, retention time, collision cross section, parent ion, and product ions with ultra-high performance liquid chromatography ion mobility quadrupole time-of-flight mass spectrometry (UPLC-IM-QTOFMS). After that, the sample preparation for milk, including extraction solvents and purified sorbents, was optimized by selecting 274 hazardous compounds as the representative compounds. Acetonitrile/methanol (9:1, *v*/*v*) containing 1% acetic acid was used for extracting, and 50 mg EMR and 50 mg PSA were used for purifying the target substances in milk. Then, the methodology was evaluated by spiking the compounds in real milk. The experiment was conducted by matrix calibration, and the results displayed that most compounds had good linearity within their linear ranges (R^2^ > 0.99). The recovery ranged from 61.8% to 119.7% at three spiking levels, with RSDs between 1.1% and 20.2%. The limits of quantitation of target compounds in milk ranged from 1 to 10 μg/kg. This could meet the MRL requirements among different countries. All the results demonstrated this determination technology was a fast, sensitive, and accurate method for screening hazardous substance.

## 1. Introduction

Milk as the key component of a balanced diet, is providing the human with abundant nutrients. The data shows that in 2023, the per capita consumption of milk in China is 41.3 kg, which is only 22.6% to 37.7% of the recommended amount in the Dietary Guidelines for Chinese Residents (2022) [1,2]. That implies a high demand for dairy and its products. The import and export of milk have been increasing. However, the safety of the milk has attracted great attention. The pesticide residue in plant products can easily enter dairy cows through feed, as well as the veterinary drugs for protecting the cows from diseases; the pesticides and veterinary drug residues remain in milk [3]. Meanwhile, due to various contaminations (e.g., moldy straw), different chemicals have been found in milk [4,5], such as mycotoxins [6], hormones, and metals. The European Union reporting on pesticide residues in foods in 2022 showed that, still, 3.7% of the sample exceeded the maximum residue levels (MRLs) [7]. Literature suggests [8,9,10,11] that different regulations of hazardous substances are considered among the exporting and importing countries that can impose trade costs.

Stricter regulations and standards on MRLs were updated [12,13] and improved by countries around the world to ensure the quality and safety of milk. In particular, Japan’s Positive List System not only imposes strict MRLs on most pesticides and veterinary drugs but also adheres to the “a zero MRL tolerance” principle [14], under which any substance not explicitly listed is subject to an MRL of 0.01 mg/kg. In recent years, China’s national food safety standards have also undergone continuous updates. However, there is still a gap compared to the food safety regulations of major trading partners. For pesticides, there are some pesticides with MRLs in the EU but not in China, and there are other pesticides with MRLs in China but not in EU. These inconsistent regulations result in heavy testing tasks during the import and export trade process. There are many methods to determine pesticides [15,16], veterinary drugs [3,17,18,19,20,21], and toxins [22,23] in milk all over the world. Manjusha R. Jadhav [14] reported a unified sample preparation approach for high-throughput 78 drugs and 238 pesticides complied with CD 2002/657/EC and SANTE/11813/2017 guidelines. Runjia Shi [18] investigate 61 veterinary drugs in commercial liquid milk products in China including imported brands and found all drug residue levels were far below the regulated maximum residue limits. Lidija Kenjeric [24] developed and validated an HPLC-MS/MS multi-class method to detect 15 different classes of veterinary drugs in milk and applied the method to real commercial milk and found no residues of veterinary drugs in the milk samples. Kanchan Yadav [23] developed a multiplexed and ultrasensitive detection of aflatoxin B1 (AFB1) and M1 (AFM1) in milk employing PEG@Mn0.02Ta3S6 NSs with TAMRA dye-labeled AFB1 aptamer and a FAM dye-labeled AFM1 aptamer, respectively. Sample pre-treatment is a critical step in the analysis. To date, there have been many clean-up techniques used in food matrices, including solid phase extraction (SPE), solid-phase microextraction (SPME) [25], liquid–liquid extraction (LLE), and QuEChERS (Quick, Easy, Cheap, Effective, Rugged and Safe). The QuEChERS approach offers advantages of efficiency, speediness, simplicity, accuracy, and good reproducibility. It is widely used for determining pesticide residues, various types of residues present in food sample [24,26]. Xueting Zhu [27] combined the QuEChERS extraction methodology with HPLC-MS/MS to sensitively detect and quantify multi-residue pesticides and veterinary drug residues in milk, revealing the presence of penbuterol in 14 samples and progesterone in 10 analyzed samples. Ideal multiclass analytical method is run in one injection [24]. Choosing simple and easy to operate sample preparation could improve efficiency especially for screening. Therefore, QuEChERS is used to purify the hazardous substances with regulatory differences for screening in milk in this study. Although there are kinds of methods to analysis pesticides and veterinary drugs, it is still short of screening hazardous substances with regulatory differences for import and export commodity trades.

To address these issues, it is imperative to establish a high-throughput detection method for hazardous substances based on regulatory differences. High-resolution mass spectrometry due to its advantages of quality accuracy, high throughput, and high scanning speed was applied in the field of trace analysis. Therefore, this study aims to construct a high-resolution mass spectrometry database for hazardous substances with regulatory differences, such as pesticides, veterinary drugs, fungal toxins, and environmental endocrine disruptors. By optimizing sample preparation, a rapid screening of hazardous substances in milk can be achieved.

## 2. Materials and Methods

### 2.1. Chemicals and Reagents

Pesticides (351), veterinary drugs (87), and toxins (24) were used in this study. Due to the large quantity of drugs involved, it is easy to cause waste by purchasing solid standard for individual compound. At the same time, in order to reduce the errors during preparation, we purchased mix standards for pesticides, veterinary drugs from Alta Technology Co., Ltd. (Tianjin, China), and the standards’ purity is all greater than 95%. The standards of toxins were from Dr. Ehrenstorfer GmbH (Augsburg, Germany) and Sigma-Aldrich (St. Louis, MO, USA). The stock solutions of the solid standards were dissolved by methanol (MeOH) with a concentration of 1000 μg/mL and stored at −20 °C. The 1 μg/mL working standard solutions were diluted by MeOH/H_2_O (1:1, *v*/*v*). Milk is commercially available from supermarket or the Internet, marked as an import and export commodity. HPLC-MS-grade MeOH, Acetonitrile (ACN), and H_2_O were purchased from Honeywell (Augsburg, Germany). Leucine enkephalin (LE), ammonium acetate, formic acid, and acetic acid (purity > 99%) were from Sigma-Aldrich (St. Louis, MO, USA). Sodium chloride (NaCl) of analytical purity was obtained from the Beijing Chemical Reagent Company (Beijing, China). The Discovery ^®^DSC-18, primary-secondary amine (PSA), graphitized carbon black (GCB), enhanced matrix removal (EMR) sorbents were from Supelco (Bellefonte, PA, USA). PSA 50 mg/MgSO_4_ 150 mg was from Dikma. Solid phase extraction (SPE) prime HLB and lipid EMR were obtained from Waters Corp (Milford, MA, USA) and Agilent Corp (Santa Clara, CA, USA), respectively. Ultrapure water was obtained from an in-house Milli-Q^®^ Ultrapure water system (Millipore, Bedford, MA, USA).

### 2.2. UPLC-IM-QTOF MS Conditions

Ultra-high performance liquid chromatography ion mobility high-resolution mass spectrometry (UPLC-IM-QTOFMS) (Waters ACQUITY^TM^, Vion, Milford, MA, USA) equipped with an ESI source was used in this experiment. Chromatographic separation was conducted on a BEH C18 column (2.1 mm × 100 mm; 1.7 μm; Waters) at 50 °C. The flow rate was 0.45 mL/min, and the injection volume was 2 μL. The gradient conditions are shown in Table 1 with the phase A being 0.1% formic acid in ACN for the positive mode and 5 mM ammonium acetate in ACN for the negative mode, and phase B was 0.1% formic acid in H_2_O for positive mode and 5 mM ammonium acetate in H_2_O for negative mode. The MS parameters were set as follows: source temperature, 120 °C; capillary voltage, 1.0 kV; desolvation temperature, 450 °C; desolvation gas rate, 800 L/h. The *m*/*z* range was 50–1500 Da. The acquisition mode was HDMS^E^ with a low energy of 6 eV and elevated energy ramping from 10 to 45 eV to obtain the protonated or deprotonated molecule and the product ions for the compound in one injection. To ensure the accuracy of mass, the real-time calibration of LE (50 ng/mL, positive ion mode: 556.2771) was carried out. The standard was acquired by UPLC-IM-TOF MS with ESI positive mode and negative mode. The data were processed by UNIFI 1.7 software (Waters Corp.). Statistical analysis was determined using in SPSS Statistics 26.0 software, and graphs were generated using Origin 2018 software.

### 2.3. Sample Preparation

Fresh and drug-free milk samples were used as negative controls and stored at 4 °C before analysis. First, weight 2.0 g of homogenized liquid milk into a 15 mL centrifuge tube, and add 4 mL of acetonitrile/methanol (9:1, *v*/*v*) containing 1% acetic acid, vortex for 30 s, and sonicate for 10 min, centrifuge for 10,000 rpm at 4 °C. Next, dilute the supernatant to 6 mL with acetonitrile/methanol (9:1, *v*/*v*) containing 1% acetic acid. Finally, take 1 mL of supernatant and add it into a 2 mL centrifuge tube containing 50 mg EMR and 50 mg PSA, vortex for 30 s, centrifuge for 10,000 rpm at 4 °C, and the upper layer was filtrated and then directly subjected to UPLC-IM-QTOFMS.

## 3. Results and Discussion

### 3.1. Database Construction for Hazardous Substances with Regulatory Differences

We conducted a detailed comparison between China’s current effective national food safety standards and relevant regulations of major trading countries regarding the MRLs for hazardous substances. The regulatory requirements of pesticide residues’ MRLs were compared among various countries and regions, including “National Food Safety Standard Maximum Residue Limits for Pesticides in Food of China” (GB 2763-2019) [28], Japan’s positive list, EU/EC Directive Regulations, and the Australia New Zealand Food Standards Code. Based on the collection of relevant food safety regulations between China and major trading countries, the differences MRLs of hazardous substances between them were analyzed using UPLC-IM-QTOFMS. The database included 462 regulatory differences in hazardous substances.

**Figure 1:**
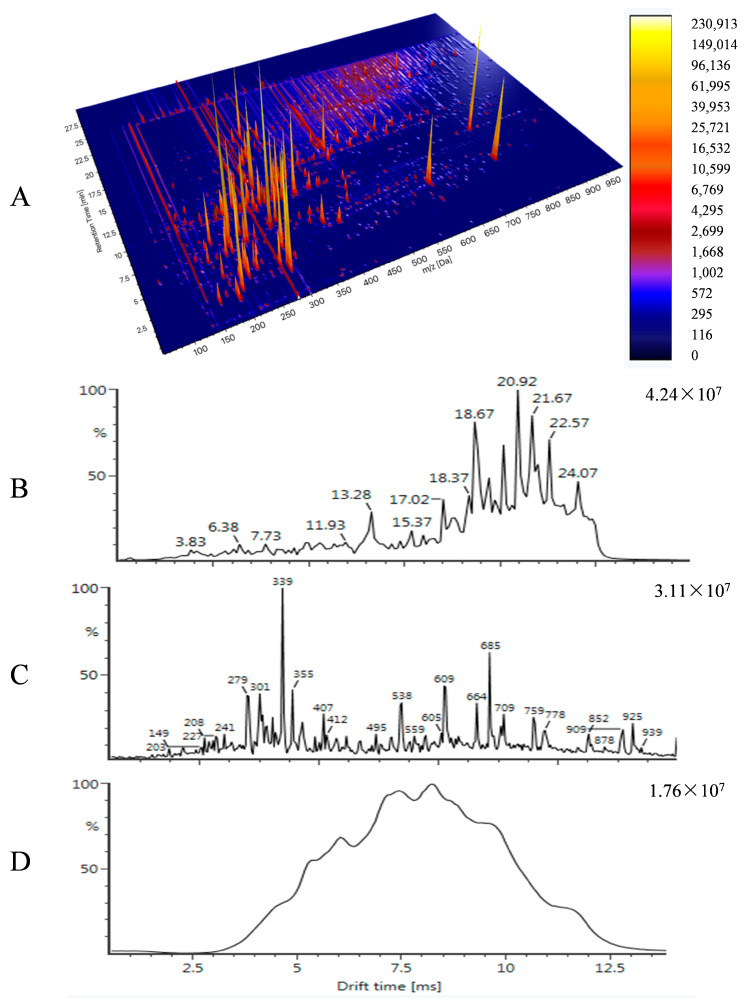
High-resolution mass spectra of 462 regulatory differentiated hazardous substances. (**A**) Three-dimensional chromatogram; (**B**) total ion chromatogram; (**C**) mass distribution; (**D**) drift time distribution.

The standard was injected into UPLC-IM-QTOFMS, and we obtained the full chromatography. Combined with the MOL molecular information of each compound, the parent ion information of the compound is automatically identified using UNIFI 1.7 data processing software, and relevant information such as retention time, fragment information, collision cross section (CCS), etc., were obtained (Supplement Table S1). Quality control (QC) and LE also were used to investigate the sensitivity and accuracy of the instrument in the whole experiment. The CCS is calculated by drift time and its value represents a compound specific physiochemical descriptor [29]. We compared the CCS we obtained with the CCS from Zhulab’website (http://allccs.zhulab.cn/, accessed on 2 January 2025) [30] and found that CCS is not affected by the chromatography condition and instruments, which could serve as a valuable additional identification parameter. The literature [30] displayed that compared with retention time (RT) + MS/MS, RT + MS/MS + CCS could sharply reduce the candidate substances effectively when screening. Therefore, in this study, we add that the CCS values of the compounds accompany with RT and product ions in the database. All 462 compounds were mixed into a mixed standard with a concentration of 20 ppb and injected according to the same acquired conditions. We could see 462 compounds were separated by LC and ion mobility. The three-dimensional chromatograms and spectra of 462 hazardous substances were shown in Figure 1. It showed the spectra acquired by UPLC-IM-QTOFMS from different perspectives. It might be seen that chromatography cannot completely separate them when large number of chemical substances exist in a sample. Under this circumstance, on one hand, basing on different *m*/*z* values using extracted ion chromatograms (EIC), co-eluted compounds can be separated; on the other hand, the drift time may play an important role to assistant in separation especially for distinguishing isomeric compounds, which demonstrated that CCS would be a valuable additional identification parameter for screening.

### 3.2. Optimization of Sample Preparation

#### 3.2.1. Extraction Solvent

Acetonitrile is commonly used as an extraction solvent to effectively precipitate proteins and remove interferences in milk matrix [26]. However, some pesticides and veterinary drugs formed bound states with proteins in milk in this experiment, affecting the extraction efficiency for these substances. Therefore, based on our prior experience, acetonitrile containing 1% acetic acid was used as the extraction solvent, which can not only effectively remove proteins but also convert target compounds into free state, thereby increasing the extraction efficiency of the target compounds. On the other hand, some milk, due to its production process, may experience layer separation when acetonitrile is used as the extraction solvent. Adding 10% methanol can eliminate this layer separation phenomenon and significantly improve the extraction rate of veterinary drugs [31]. So, a mixture of acetonitrile/methanol (9:1, *v*/*v*) containing 1% acetic acid was ultimately selected as the extraction solvent system in this study.

#### 3.2.2. Volume of Extraction Solvent

Furthermore, the volume ratio of the extraction solvent to milk is also an important factor affecting protein precipitation effect. Therefore, this study investigated the protein precipitation effects at volume ratios of milk to extraction solvent of 1:1, 1:2, and 1:3. The results revealed that when the volume ratio of milk to extraction solvent was 1:1, only partial protein precipitation occurred, and the supernatant remained turbid after centrifugation to remove the precipitate, indicating incomplete protein precipitation. When the volume ratios were 1:2 and 1:3, complete precipitation of milk proteins was achieved, resulting in a clear supernatant. However, considering that a volume ratio of 1:3 diluted the sample excessively and reduced the method’s sensitivity, a volume ratio of 1:2 for milk to extraction solvent was ultimately chosen as the extraction condition.

#### 3.2.3. Purified Sorbents

**Figure 2:**
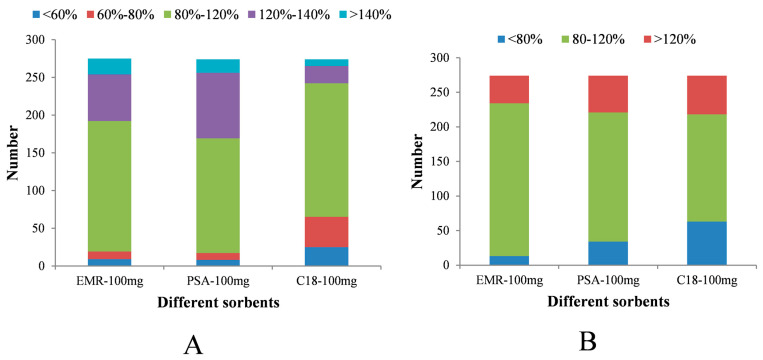
Distribution of recovery (**A**) and matrix effects (**B**) of target compounds when using EMR, PSA, and C18 as purification sorbents.

EMR, as a new type of polymeric material, can specifically remove fatty acids in the sample matrix and reduce matrix effects. So, this study compared the purification effects of EMR with traditional purification sorbents C18 and PSA. The experiment selected 274 target compounds for investigation, after centrifuging the milk extract (with a spiking concentration of 15 μg/kg), 1 mL of the supernatant was taken, and 100 mg of EMR, C18, or PSA was added, followed by vortexing for 30 s and centrifugation at 10,000 rpm for 10 min at 4 °C. The supernatant was then taken for direct analysis to evaluate the purification effects of the three materials. As shown in Figure 2A, the results found that EMR and PSA as purification sorbents yielded good recovery rates, with over 90% of the compounds having recovery rates between 60% and 140% for all target compounds. When C18 was used as the purification material, the recovery rates of 25 target compounds were all below 60%. Meanwhile, matrix effects were also an important factor in evaluating purification effects. Therefore, we also examined the matrix effects of the three purification sorbents. As shown in Figure 2B, when EMR was used as the purification material, the matrix effects for over 80% of the target compounds were between 80% and 120%, indicating the best purification effect. With PSA as the purification material, 68% of the target compounds had matrix effects between 80% and 120%, with a moderate purification effect. As shown in Figure 2B, C18 had strong matrix effects and the worst purification effect. Therefore, based on the above results, we believe that C18 is not suitable for high-throughput determination of these compounds due to its strong adsorption and matrix effects, while EMR and PSA are more suitable for simultaneous purification of multiple compounds.

**Figure 3:**
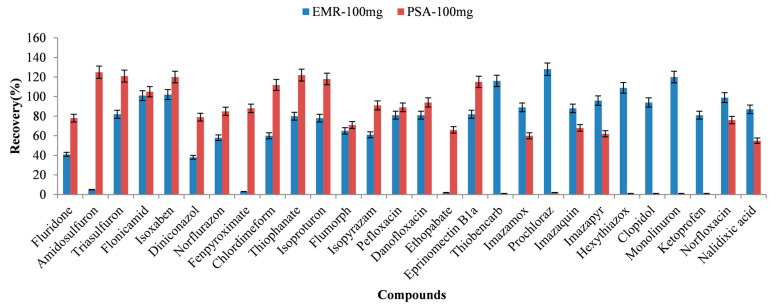
Recovery of selected target compounds when using EMR and PSA as purification sorbents.

As shown in Figure 3, EMR exhibits a better recovery for some compounds such as thiobencard, prochloraz, and hexythiazox, while PSA exhibits a better recovery for other compounds such as amidosulfuron and chlordimeform. To overcome the shortcomings of EMR or PSA, the combination of EMR and PSA was investigated for purifying these compounds.

#### 3.2.4. The Amount of Purified Sorbents

**Figure 4:**
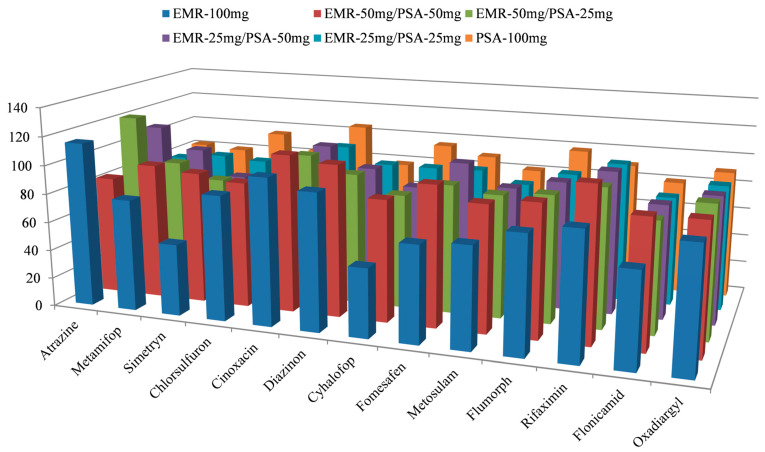
Recovery of selected target compounds when using EMR and PSA in combination for purification.

The purified sorbents 100 mg of EMR, 50 mg EMR/50 mg PSA, 50 mg EMR/25 mg PSA, 25 mg EMR/50 mg PSA, 25 mg EMR/25 mg PSA, and 100 mg of PSA was further investigated to evaluate the recovery. The results revealed that using different ratios of EMR-PSA as purification materials did not significantly differ in the distribution of recovery rates for most target compounds, and most achieved good recovery. However, for individual compounds, there were significant differences in recovery rates when using EMR and PSA as purification materials alone, whereas combining them into an EMR-PSA mixture yielded better recovery. As shown in Figure 4, when 50 mg of EMR was combined with 50 mg of PSA for purification, satisfactory recovery was obtained for all compounds. The final choice was to use 50 mg of EMR combined with 50 mg of PSA as the purification sorbent.

### 3.3. Method Validation

The method validation for high-resolution detection was carried out according to “Acceptance Criteria for Confirmation of Identity of Chemical Residues using Exact Mass Data for the FDA Foods and Veterinary Medicine Program” published by the office of foods and veterinary medicine (OFVM) of FDA in 2015 [32] and European Commission Regulation 2021/808. For confirmation, there must be at least two accurate masses (generally one precursor and one product ion), and mass deviation for precursor ion should be less than or equal to 5 ppm and product ions should be less than or equal to 10 ppm. The set for retention time is within ±2.5%, not to exceed 0.5 min.

The matrix effect (ME, %) was calculated as the ratio between the area of matrix-matched calibration solution and the area of standard solution at the same concentration under identical conditions and then multiplied by 100%. It is generally considered that the ME is ignored if the data are 80–120%. It is ME enhancement if the value is above 120% and inhibition if the value is lower than 80%. The result showed that the matrix had a significant impact on target analyte detection, so matrix-matched standards were used for quantification. According to the optimized sample preparation, the blank milk samples were processed to form the matrix solution diluted the standard working solution was diluted with the matrix solution step by step to obtain a series of matrix calibration with concentrations of 0.3, 1, 5, 10, 25, 50, 100, and 300 μg/kg. Using the peak area of the target analyte as the ordinate (Y) and the corresponding concentration as the abscissa (X), a standard curve was obtained. The lowest spiking concentration at which the signal-to-noise ratio (S/N) of the target compound was greater than 3 and 10 was considered as the limit of detection (LOD) and the limit of quantification (LOQ), respectively.

**Figure 5:**
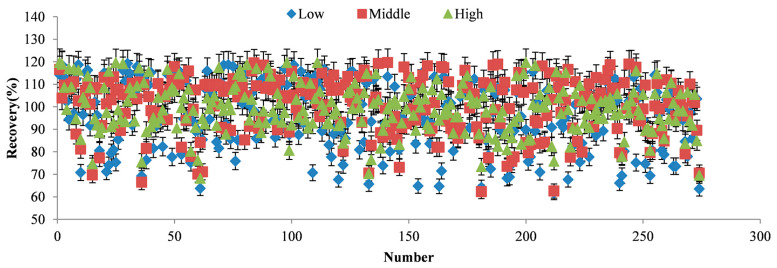
The recoveries distribution for the compounds at three spike concentrations.

The results indicated that most compounds had good linearity within their respective linear ranges (R^2^ > 0.99). The LODs for the 274 compounds ranged from 0.3 to 3.0 μg/kg, and the LOQs ranged from 1.0 to 10 μg/kg. The recovery for all compounds at three spiking levels ranged from 61.8% to 119.7% (Figure 5), with RSDs between 1.1% and 20.2%. The recovery rates and RSDs for the 274 target compounds at the three spiking levels were presented in Table S2.

In addition, the quantitative limit of this method was compared with the MRLs requirements of countries and regions. The results found that there were 138 out of 274 hazardous substances with regulatory differences in milk. According to Table S2, the LOQ for the 138 compounds in this method is less than or equal to the MRLs in different countries. Furthermore, the LOQ of this method is lower than the MRL limit requirement, and the recovery for these compounds at three concentration spiked levels were between 60 and 120%, with RSD less than 20%, which can meet the needs of international trade detection.

Meanwhile, this study compared the LOQs of the target compound in milk with national standards for food in China. The results showed that 61 out of 138 target compounds had corresponding national standard methods for determination in milk, and over 50% of the target compounds had LOQ lower than those of the national standard in China (Table 2).

### 3.4. Detection of the Real Sample

**Figure 6:**
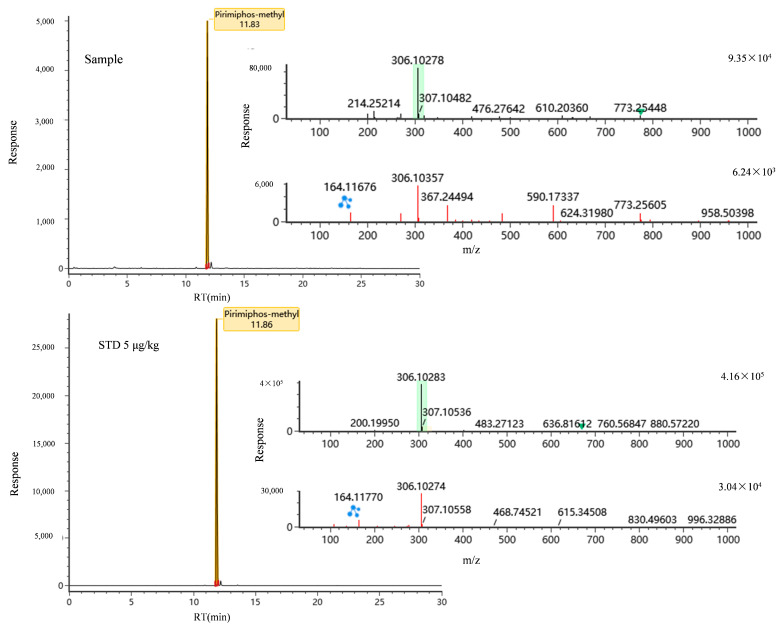
The chromatography and mass spectrum of real sample (**up**) and the standard (**down**) for pirimiphos-methyl. (Note: the green highlights in the figure means the parent ion of pirimiphos-methyl; the blue shape represents the fragment ion of pirimiphos-methyl (306.10274). The green triangle represents the cursor position. The red line has no meaning, it just shows with red color not black).

The method was applied to 32 actual liquid milk samples with different brands and production batches. The samples sourced from different origins such as Asia, Europe, Australia, and New Zealand, including Yili, Sanyuan, Junlebao, Mengniu, Weidendorf, and Theland. The samples were processed using the preparation method and acquired by UPLC-IM-QTOFMS. For screening parameters setting, at least two accurate masses are required, one is the parent ion and the other is the product ion, and mass deviation for the parent ion should be less than or equal to 5 ppm, and product ions should be less than or equal to 10 ppm. The results showed that pirimiphos-methyl was present in four actual liquid milk samples. From Figure 6 we can see the top spectrum is the MS data, while the bottom spectrum is the MS/MS data for the compound; the 164.11676 and 164.11770 highlighted in green is the product ion of the pirimiphos-methyl. As for the RT of pirimiphos-methyl, it is 11.67 min in the database, while it is 11.83 min in real sample, the error is 1.4%. It is the acceptance for screening. From the previous studies, quinolones and tetracyclines [21] were detected in milk, but in our study there were four real liquid milk samples containing pirimiphos-methyl. The concentration of pirimiphos-methyl was between 2.2 and 4.6 μg/kg and was below the MRLs for all countries, which can meet the trade requirement.

## 4. Conclusions

This study established a mass database for 462 hazardous substances with different MRLs among countries regulations using UPLC-IM-QTOFMS technology and based on QuEChERS methods quantified the compounds in milk. The database proved to be reliable for HDMS scanning mode, accurately determining the masses of parent ions, product ions and CCS value. The milk was extracted by acetonitrile/methanol (9:1, *v*/*v*) containing 1% acetic acid and purified by 50 mg EMR and 50 mg PSA. The validation procedure included assessments of matrix effect, linearity, LOD, LOQ, accuracy, and precision. The recoveries at three spiking levels ranged from 61.8% to 119.7%, with RSDs between 1.1% and 20.2%. The LOQs for these compounds range from 1 to 10 μg/kg, less than or equal to the MRLs according to the regulations, meeting the needs of international trade detection. The analytical separation and detection procedure significantly reduced analysis time and increased sample throughput. The developed method was successfully applied to analyze real samples. Out of 32 samples, 4 positive samples were detected. This method provides an integrated strategy for effectively screening hazardous substances with regulatory differences in milk for exporting and importing trade.

## Figures and Tables

**Table 1:** The gradient condition of mobile phase of UPLC.

Time (min)	%A	%B
0	2	98
0.5	2	98
20	99	1
24	99	1
25	2	98
30	2	98

**Table 2:** Comparison of LOQ of target compounds with current effective national standard quantification limits in China.

Compound	LOQ in This Method (μg/kg)	LOQ in National Standard in China (μg/kg)	Reference Method
Ametryn	1	12.5	GB/T 23210-2008 Determination of 511 pesticides and related chemicals residues in milk and milk power-GC-MS method [33]
Anilofos	1	8.3	
Atrazine	1	4.2	
Benalaxyl	1	4.2	
Buprofezin	1	8.3	
Carfentrazone-ethyl	1	8.3	
Chlordimeform	1	16.8	
Cloquintocet-mexyl	1	4.2	
Cyanazine	1	12.5	
Cyproconazole	1	4.2	
Diazinon	1	4.2	
Dimethachlor	1	12.5	
Diniconazol	1	12.5	
Edifenphos	1	8.3	
Etoxazole	1	25	
Fenamidone	1	16.8	
Fenamiphos	1	12.5	
Fenarimol	1	8.3	
Fenpropimorph	1	4.2	
Fenpyroximate	1	33.3	
Fluazifop-butyl	1	4.2	
Flusilazole	1	12.5	
Halosulfuron methyl	1	333.2	
Hexazinone	1	12.5	
Metribuzin	1	12.5	
Myclobutanil	1	4.2	
Napropamide	1	12.5	
Norflurazon	1	4.2	
Pirimicarb	1	8.3	
Pirimiphos-ethyl	1	8.3	
Pirimiphos-methyl	1	4.2	
Propyzamide	1	8.3	
Spiroxamine	1	8.3	
Acrinathrin	5	8.3	
Boscalid	5	10	
Butamifos	5	4.2	
Ciprofloxacin	5	10	
Clodinafop-propargyl	5	8.3	
Enrofloxacin	5	10	
Fenhexamid	5	83.3	
Fluquinconazole	5	4.2	
Acrinathrin	5	8.3	
Boscalid	5	10	
Butamifos	5	4.2	
Ciprofloxacin	5	10	
Clodinafop-propargyl	5	8.3	
Enrofloxacin	5	10	
Fenhexamid	5	83.3	
Fluquinconazole	5	4.2	
Profenofos	5	10	
Pyraflufen-ethyl	5	8.3	
Pyridaben	5	4.2	
Thiabendazole	5	30	
Tricyclazole	5	25	
Zoxamide	5	8.3	
Tribenuron-methyl	10	12	
Diuron	1	1.2	GB/T 23211-2008 Determination of 493 pesticides and related chemicals residues in milk and milk power-LC-MS-MS method [34]
Furathiocarb	1	1.5	
Isoprothiolane	1	1.35	
Pyrazophos	1	1.2	
Omethoate	5	7	
Sulfentrazone	5	22.4	
Cyhalofop	10	25	
Triallate	10	15	
Doramectin	1	2	GB 29696-2013 Determination of Avermectins residues in milk by High Performance Liquid Chromatographic method [35]
Difenoconazole	1	5	GB 23200.49 Determination of difenoconazole residue in foods Gas chromatography-mass spectrometry [36]
Quinoxyfen	1	3	GB 23200.56 Determination of quinoxyfen residue in foods [37]
Sarafloxacin	5	10	GB 29692-2013 Determination of quinolones residues in milk by High Performance Liquid Chromatographic method [38]
Zearalenone	1	4	GB 5009.209-2016 Determination of zearalenone in foods [39]

## Data Availability

The original contributions presented in the study are included in the article/Supplementary Materials, and further inquiries can be directed to the corresponding author.

## Supplementary Materials

The following supporting information can be downloaded at: https://www.mdpi.com/article/10.3390/foods14060967/s1, Table S1. The mass database of hazardous substances with regulatory differences. Table S2. Quantitative limit, recovery, and RSD of harmful substances in milk due to regulatory differences and MRL in different countries and regions.
