# Supplementary material for: Rapid Screening for Hazardous Substances with Regulatory Differences in Milk Between Countries Using Ultra-High Performance Liquid Chromatography Ion Mobility Quadrupole Time-of-Flight Mass Spectrometry
Source: Foods. 2025 Mar 12;14(6):967. doi: 10.3390/foods14060967 (PMC11941072; doi:10.3390/foods14060967)
Supplement: Supplementary file 1 [file foods-14-00967-s001.zip › foods-3462882-supplementary.pdf]

Rapid Screening for Hazardous Substances with Regulatory Differences in Milk Between Countries Using Ultra-High Performance Liquid Chromatography Ion Mobility Quadrupole Time-of-Flight Mass Spectrometry

Qiaozhen Guo [1,2], Jing Zhang [1,2], Bing Shao [1,2], Jie Yin [1,2], Yunjia Yang [1] and Yi Yang [1,2,*]

[1] Key Laboratory of Diagnostic and Traceability Technologies for Food Poisoning, Beijing Center for Disease Control and Prevention, Beijing 100013, China; qiao037@126.com (Q.G.); brightjing@163.com (J.Z.); shaobingch@sina.com (B.S.); arnold_jay@163.com (J.Y.); greendayang@163.com (Y.Y.)

[2] School of Public Health and Family Medicine, Capital Medical University, Beijing 100089, China

* Correspondence: yangyi@bjcdc.org

Table S1. The mass database of hazardous substances with regulatory differences

Category	Name	CAS	Molecular Formula	Neutral mass (Da)	Observed m/z	Mass error (ppm)	RT (min)	CCS	Fragments	Adducts	Mode
Veterinary drug	Sulfameter	651-06-9	C11H12N4O3S	280.0630	281.0687	-3.61	3.88	159.04	156.01138, 108.0454, 215.0935, 126.06619	+H, +Na	ESI+
Veterinary drug	Sulfapyridine	144-83-2	C11H11N3O2S	249.0572	250.0629	-4.02	2.68	151.57	156.01138, 184.0871, 108.0438, 95.06062	+H, +Na	ESI+
Veterinary drug	Sulfabenzamide	127-71-9	C13H12N2O3S	276.0568	277.0622	-4.93	4.99	157.96	156.0115,108.0461,92.0494	+H, +Na	ESI+
Veterinary drug	Sulfamonomethoxine	1220-83-3	C11H12N4O3S	280.063	281.0703	2.14	3.37	158.94	188.0124, 156.01138, 126.06619, 92.04948	+H	ESI+
Veterinary drug	Sulfamethoxazole	723-46-6	C10H11N3O3S	253.0521	254.0579	-3.60	4.24	151.62	156.01138,188.0818 92.04948,	+H, +Na	ESI+
Veterinary drug	Sulfadiazine	68-35-9	C10H10N4O2S	250.0524	251.0581	-4.20	2.22	150.8	156.01138, 92.04948, 96.05562	+H, +Na	ESI+
Veterinary drug	Sulfaphenazole	526-08-9	C15H14N4O2S	314.0837	315.0900	-1.43	5.46	169.1	156.01138, 160.08692, 222.03317	+H, +Na	ESI+
Veterinary drug	Sulfamethozol	144-82-1	C9H10N4O2S2	270.0245	271.0320	2.96	3.35	154.21	108.0447,92.0498,156.01138	+H	ESI+
Veterinary drug	Sulfamethazine	57-68-1	C12H14N4O2S	278.0837	279.0909	1.62	3.47	161.24	186.03317, 124.0871, 108.0445, 92.05037	+H, +Na	ESI+
Veterinary drug	Sulfacetamide	144-80-9	C8H10N2O3S	214.0412	237.0301	-3.78	1.79	152.09	156.01138	+Na, +H	ESI+
Veterinary drug	Sulfathiazole	72-14-0	C9H9N3O2S2	255.0136	256.0193	-3.92	2.5	150.88	156.01138, 92.04948,108.0444	+H, +Na	ESI+
Veterinary drug	Sulfamethoxypyridazine	80-35-3	C11H12N4O3S	280.0630	281.0693	-1.46	3.63	159.98	126.0662, 156.01138, 92.04948, 111.04271	+H, +Na	ESI+
Veterinary drug	Sulfisoxazole	127-69-5	C11H13N3O3S	267.0677	268.0732	-4.72	4.61	159.11	108.1459, 156.01138, 92.04948	+H, +Na	ESI+
Veterinary drug	Sulfanitran	122-16-7	C14H13N3O5S	335.0575	336.0638	-1.46	6.77	171.89	294.0543, 134.06004, 198.02194	+H, +Na	ESI+
Veterinary drug	Sulfadoxine	2447-57-6	C12H14N4O4S	310.0735	311.0796	-2.19	4.46	166.04	156.01138, 154.0611, 141.05328, 92.04948	+H, +Na	ESI+
Veterinary drug	Sulfamerazine	127-79-7	C11H12N4O2S	264.0681	265.0742	-2.27	2.87	156.16	172.01752, 156.01138, 92.04948	+H, +Na	ESI+
Veterinary drug	Sulfachinoxalin	59-40-5	C14H12N4O2S	300.0681	301.0742	-2.00	5.54	165.97	108.0444, 146.07162, 156.0113, 129.04472	+H, +Na	ESI+
Veterinary drug	Sulfachloropyridazine	80-32-0	C10H9ClN4O2S	284.0134	285.0193	-3.06	3.94	158.03	156.01138, 92.04948, 108.0457	+H, +Na	ESI+
Veterinary drug	Sarafloxacin	98105-99-8	C20H17F2N3O3	385.1237	386.1311	1.82	4.35	201.66	299.0991, 342.14125, 285.0834, 271.07516	+H, +Na	ESI+
Veterinary drug	Griseofulvin	126-07-8	C17H17ClO6	352.0713	353.0772	-2.47	8.1	180.83	285.0524, 165.05462, 321.0524, 278.03404	+H, +Na	ESI+
Veterinary drug	Sparfloxacin	110871-86-8	C19H22F2N4O3	392.166	393.1728	0.25	4.52	199.02	349.1834, 292.1256, 251.08647, 318.10486	+H, +Na	ESI+

Veterinary drug	Betamethasone	378-44-9	C22H29FO5	392.1999	415.1883	-3.31	3.64	221.6	355.1911,89.05971	+Na, +H	ESI+
Veterinary drug	Metoclopramide	364-62-5	C14H22ClN3O2	299.14005	300.1460	-2.51	3.95	173.41	227.05818, 184.01598, 212.03471	+H, +Na	ESI+
Veterinary drug	Mecillinam	32887-01-7	C15H23N3O3S	325.14601	326.1524	-0.95	6.16	178.14	160.04268, 199.05357	+H, +Na	ESI+
Veterinary drug	Moxidectin	113507-06-5	C37H53NO8	639.37712	640.3848	1.53	16.25	262.49	622.3738, 548.30066, 121.0886, 549.28468	+Na, +H	ESI+
Veterinary drug	Kitasamycin	1392-21-8	C35H59NO13	701.39864	702.4067	1.94	6.26	308.28	174.11247,540.31671	+H, +Na	ESI+
Veterinary drug	Troleandomycin	2751-09-9	C41H67NO15	813.45107	814.4584	0.77	8.77	305.68	174.11247,582.32727,109.06479,796.4478	+H, +Na	ESI+
Veterinary drug	Maduramicin	84878-61-5	C47H83NO17	933.5661	934.5750	2.36	14	305.98		+H	ESI+
Veterinary drug	Robenidine	25875-51-8	C15H13Cl2N5	333.0548	334.0611	-1.20	9.29	186.13	195.0432,138.0105,155.03705,110.9996	+H	ESI+
Veterinary drug	Salinomycin	53003-10-4	C42H70O11	772.47376	773.4814	1.22	14.24	277.08	644.46466,255.15909,267.1227	+H, +Na	ESI+
Veterinary drug	Clenbuterol	37148-27-9	C12H18Cl2N2O	276.07962	277.0854	-3.33	4.38	165.94	168.04488,203.0140,259.07633,185.98718	+H	ESI+
Veterinary drug	Spiramycin	24916-50-5	C43H74N2O14	842.51401	843.5220	1.53	5.63	330.01	174.11247,699.44264,684.39535,522.3061	+H, +Na	ESI+
Veterinary drug	Tulobuterol	41570-61-0	C12H18ClNO	227.10769	228.1137	-3.04	4.43	152.41	154.0418,119.07295,117.0573	+H	ESI+
Veterinary drug	Chlormadinone acetate	302-22-7	C23H29ClO4	404.17544	405.1815	-1.58	11.11	199.39	267.17434,309.18491,265.15869,345.1615	+H	ESI+
Veterinary drug	Ethopabate	59-06-3	C12H15NO4	237.10011	260.0891	-2.99	5.52	152.45	137.02332,178.04987,147.04406,178.0862	+Na, +H	ESI+
Veterinary drug	Doramectin	117704-25-3	C50H74O14	898.50786	921.4988	1.38	15.74	305.46	881.50457,331.22677,575.33672,313.2162	+Na, +H	ESI+
Veterinary drug	Cefoperazone	62893-19-0	C25H27N9O8S2	645.1424	668.1315	-0.93	4.54	238.17	530.13401,148.0393,360.06487,410.04279	+Na, +H	ESI+
Veterinary drug	Tylosin B	11032-98-7	C39H65NO14	915.51915	916.5280	2.35	7.88	318.81	174.11247,598.35857	+H, +Na	ESI+
Veterinary drug	Methylprednisolone	83-43-2	C22H30O5	374.20932	397.1976	-3.79	7.1	189.29	185.09609,253.15869,237.12739,357.2060	+Na, +H	ESI+
Veterinary drug	Ketoprofen	22071-15-4	C16H14O3	254.09429	255.1011	0.43	8.46	156.31	177.05462,77.03858,105.03349	+H, +Na	ESI+
Veterinary drug	Rafoxanide	22662-39-1	C19H11Cl2I2NO3	624.82053	625.8273	0.11	16.93	215.96	127.01833,253.00557	+H	ESI+
Veterinary drug	Eprinomectin B1a	123997-26-2	C50H75NO14	913.51876	936.5089	0.48	14.46	310.82	112.07569,490.27993,860.49434,154.0862	+Na, +H	ESI+
Veterinary drug	Altrenogest	850-52-2	C21H26O2	310.19328	311.2007	2.32	9.72	176.38	293.18999,251.14304,269.15361,199.1117	+H, +Na	ESI+
Veterinary drug	Decoquinate	18507-89-6	C24H35NO5	417.25152	418.2593	2.59	13.73	216.13	204.02913,232.06043,206.04478,344.1856	+H, +Na	ESI+
Veterinary drug	Monensin	17090-79-8	C36H62O11	670.42921	693.4178	-1.66	7.87	240.8	675.4081	+Na, +H	ESI+
Veterinary drug	Tolfenamic acid	13710-19-5	C14H12ClNO2	261.05566	262.0623	-0.23	11.74	154.11	244.052551, 209.082886	+H	ESI+

Veterinary drug	Tripelennamine	91-81-6	C16H21N3	255.17355	256.1802	-0.20	5.06	158.17	211.1215	+H	ESI+
Veterinary drug	Oxolinic acid	14698-29-4	C13H11NO5	261.06372	262.0696	-3.14	5.19	149.75	216.02913,214.01348,215.02131,216.0655	+H, +Na	ESI+
Veterinary drug	Nicarbazine	330-95-0	C19H18N6O6	426.1287	427.1368	3.29	9.66	243		+H	ESI+
Veterinary drug	Clorsulon	60200-06-8	C8H8Cl3N3O4S2	378.9021	401.8931	3.43	13.81	180	312.15942.294.14886.337.20363.284.1645	+Na	ESI+
Veterinary drug	Tiopronin	1953-02-2	C5H9NO3S	163.0303	164.0365	-3.07	2.72	137.6	76	+H	ESI+
Veterinary drug	Azaperone	1649-18-9	C19H22FN3O	327.17469	328.1813	-0.28	4.23	179.37	123.02407,95.02915,147.09167,149.10732	+H, +Na	ESI+
Veterinary drug	Diminazene	536-71-0	C14H15N7	281.1388	282.1462	2.49	2	164.14	135.0791,103.04165,91.04165	+H	ESI+
Veterinary drug	Flubendazole	31430-15-6	C16H12FN3O3	313.08627	314.0933	1.05	6.87	173.64	95.02915,123.02407,186.0298,254.07242	+H, +Na	ESI+
Veterinary drug	Oxibendazole	20559-55-1	C12H15N3O3	249.11134	250.1175	-2.17	5.36	157.81	218.0924,148.05054,175.03763,130.03997	+H, +Na	ESI+
Veterinary drug	Sulfaquinoxaline	59-40-5	C14H12N4O2S	300.068	301.0760	4.33	5.7	164.25	144.05562,146.07127,156.01138,129.0447	+H	ESI+
Veterinary drug	Danofloxacin	112398-08-0	C19H20FN3O3	357.14887	358.1559	0.92	3.72	190.6	340.1483,82.0651,255.0578	+H, +Na	ESI+
Veterinary drug	Flumequine	42835-25-6	C14H12FNO3	261.08012	262.0866	-0.84	6.79	150.93	244.0764	+H, +Na	ESI+
Veterinary drug	Gamithromycin	145435-72-9	C41H78N2O12	776.53983	777.5489	3.05	6.15	287.9		+H, +Na	ESI+
Veterinary drug	Thiabendazole	148-79-8	C10H7N3S	201.03607	202.0434	3.13	2.83	142.41	175.0332,143.06037	+H, +Na	ESI+
Veterinary drug	Monepantel	887148-69-8	C20H13F6N3O2S	473.06327	474.0704	0.91	12.43	208.27	228.06307,203.06152	+H, +Na	ESI+
Veterinary drug	Halofuginone	55837-20-2	C16H17BrClN3O3	413.01418	414.0209	0.05	5.44	190.06	100.0771	+H, +Na	ESI+
Veterinary drug	5-Hydroxy Flunixin	75369-61-8	C14H11F3N2O3	312.07218	313.0787	-0.58	7.98	167.26	252.0505,275.06266,226.07368	+H, +Na	ESI+
Veterinary drug	Rifaximin	80621-81-4	C43H51N3O11	785.35236	786.3605	1.83	10.24	275.08	151.0759,754.3421	+H, +Na	ESI+
Veterinary drug	Albendazole oxide	54029-12-8	C12H15N3O3S	281.08341	282.0892	-3.24	4.02	167.02	159.04271,191.01478,240.04374,207.0097	+H, +Na	ESI+
Veterinary drug	Morantel	20574-50-9	C12H16N2S	220.1034	221.1108	3.18	4.46	155.88	123.0263,149.02937,205.0794,150.0372	+H	ESI+
Veterinary drug	Penicillin G procaine	6130-64-9	C29H40N4O7S	334.09873	335.1050	-1.29	2.33	158.52		+H	ESI+
Veterinary drug	Enrofloxacin	93106-60-6	C19H22FN3O3	359.16452	360.1711	-0.33	3.84	195.75	245.10847,203.06152,314.16632,189.0458	+H, +Na	ESI+
Veterinary drug	Sulfaguanidine	57-67-0	C7H10N4O2S	214.0524	215.0597	2.80	0.92	150.84	156.0115,92.04948	+H	ESI+
Veterinary drug	Carazolol	57775-29-8	C18H22N2O2	298.16813	299.1744	-1.44	5.19	169.1	184.07569,196.07569,168.08078,167.0729	+H, +Na	ESI+
Veterinary drug	Tobramycin	32986-56-4	C18H37N5O9	467.25913	468.2660	0.36	0.46	163.1	324.17653,187.10772,145.09715,329.1819	+Na, +H	ESI+

Veterinary drug	Ofloxacin	82419-36-1	C18H20FN3O4	361.14378	362.1508	0.89	3.39	189.08	318.1608,261.1024	+H, +Na	ESI+
Veterinary drug	Pefloxacin	70458-92-3	C17H20FN3O3	333.14887	334.1552	-1.11	3.56	192.16	290.1659,233.1082,316.1451	+H, +Na	ESI+
Veterinary drug	Nalidixic acid	389-08-2	C12H12N2O3	232.08479	233.0905	-4.27	6.68	144.41	215.3,233.0921	+H, +Na	ESI+
Veterinary drug	Pipemidic acid	51940-44-4	C14H17N5O3	303.13314	304.1393	-1.78	2.88	174.83	217.1085,260.1507	+H, +Na	ESI+
Veterinary drug	Ciprofloxacin	85721-33-1	C17H18FN3O3	331.13322	332.1399	-0.06	3.62	190.7	149.0583,288.2478	+H, +Na	ESI+
Veterinary drug	Cinoxacin	28657-80-9	C12H10N2O5	262.05897	263.0648	-3.32	4.82	148.01	189.0278,217.0589	+H, +Na	ESI+
Veterinary drug	Enoxacin	74011-58-8	C15H17FN4O3	320.12847	321.1345	-2.09	3.35	179.86	250.063,206.0825	+H, +Na	ESI+
Veterinary drug	Norfloxacin	70458-96-7	C16H18FN3O3	319.13322	320.1393	-1.94	3.47	179.31	302.1311,233.1082	+H, +Na	ESI+
Veterinary drug	Gatifloxacin	112811-59-3	C19H22FN3O4	375.15943	376.1661	-0.08	4.33	196.47	315.1332,290.1261	+H, +Na	ESI+
Veterinary drug	Fleroxacin	79660-72-3	C17H18F3N3O3	369.13003	370.1366	-0.35	3.46	192.14	269.0908,326.1473,222.0603	+H, +Na	ESI+
Veterinary drug	Butopamine	66734-12-1	C18H23NO3	301.16779	302.1735	-3.29	3.64	172.82	121.06479,164.10699,107.04914,91.05423	+H	ESI+
Veterinary drug	Monensin	1174269-36-3	C36H62O11	670.42921	669.4220	2.22	15.96	246.55	637.3957,455.3014,593.4058,153.0921	-H	ESI-
Veterinary drug	Spiramycin	8025-81-8	C43H74N2O14	842.51401	841.5047	-0.72	6.24	305.25	488.3017,823.496,474.286414.26498	-H	ESI-
Veterinary drug	Toltrazuril	69004-03-1	C18H14F3N3O4S	425.06571	424.0587	3.98	11.72	204.66	324.01858	-H	ESI-
Veterinary drug	Diminazene aceturate	908-54-3	C14H15N7	281.13889	280.1312	3.59	2.35	174.25	252.12547	-H	ESI-
Veterinary drug	Nitroxynil	1689-89-0	C7H3IN2O3	289.91883	288.9111	3.35	5.05	151.67	126.90502162.00709258.91357	-H	ESI-
Pesticide	Propyzamide	23950-58-5	C12H11Cl2NO	255.02177	256.0288	1.29	9.67	158.86	172.9555, 144.96063, 189.9821, 108.98395	+H	ESI+
Pesticide	Amitraz	33089-61-1	C19H23N3	293.1891	294.1965	2.39	12.95	163.12	132.08078,163.122	+H, +Na	ESI+
Pesticide	Propiconazole	60207-90-1	C15H17Cl2N3O2	341.06978	342.0772	2.11	10.95	177.34	186.9712, 204.98176, 273.0443, 144.96063	+H, +Na	ESI+
Pesticide	Metosulam	139528-85-1	C14H13Cl2N5O4S	417.00653	418.0139	1.61	7.63	187.07	140.02615, 173.98718, 179.05635	+H, +Na	ESI+
Pesticide	Mebendazole	31431-39-7	C16H13N3O3	295.09569	296.1032	2.74	6.35	173.21	186.0298, 77.03858, 105.03349, 159.04271	+H, +Na	ESI+
Pesticide	Terbutryn	886-50-0	C10H19N5S	241.13612	242.1435	2.82	7.8	161.02	158.04949,138.07742,144.05899,68.02432	+H	ESI+
Pesticide	Triflumizole	99387-89-0	C15H15ClF3N3O	345.0855	346.0929	2.03	8.62	188	178.98699,160.99641	+H	ESI+
Pesticide	Tepraloxydim	149979-41-9	C17H24ClNO4	341.13939	342.1467	1.79	9.97	185.52	166.08626,222.14886,252.15942,149.0597	+H	ESI+
Pesticide	Diuron	330-54-1	C9H10Cl2N2O	232.01702	233.0239	0.78	7.62	149.06	125.00268,72.04439,152.99759	+H	ESI+

Pesticide	Isouron	55861-78-4	C10H17N3O2	211.132	212.1380	-3.32	6.22	150.6	124.07569,106.06513,72.04439	+H	ESI+
Pesticide	Chlorfenapyr	122453-73-0	C15H11BrClF3N2O	405.96954	406.9768	1.38	7.55	186.4	59.0491,327.051	+H	ESI+
Pesticide	Quinoclamine	2797-51-5	C10H6ClNO2	207.00871	208.0159	2.37	5.81	134.72	77.03858,105.03349	+H	ESI+
Pesticide	Isoxaben	82558-50-7	C18H24N2O4	332.17361	355.1634	0.27	10.33	185.38	150.03115,122.03623,107.01276	+Na, +H	ESI+
Pesticide	Dazomet	533-74-4	C5H10N2S2	162.0285	163.0358	3.70	6.56	125.93	119.9934,90.03703	+H	ESI+
Pesticide	Chloridazon	1698-60-8	C10H8ClN3O	221.0355	222.0429	3.17	4.14	142.36	146.01157,77.03858,186.06619,205.01632	+H	ESI+
Pesticide	Chlorfluazuron	71422-67-8	C20H9Cl3F5N3O3	538.96297	539.9693	-0.69	14.44	219.89	346.95964,141.01465,186.95862,158.0412	+H	ESI+
Pesticide	Ivermectin	71827-03-7	C48H74O14	874.50786	897.4969	-0.75	17.03	297.54	307.22677,567.33163	+Na	ESI+
Pesticide	Linuron	330-55-2	C9H10Cl2N2O2	248.01193	249.0194	3.10	9.03	151.77	160.97936,216.99299,159.97153,181.0163	+H	ESI+
Pesticide	Fenarimol	60168-88-9	C17H12Cl2N2O	330.0326	331.0400	2.12	9.64	173.79	278.06053,295.06327,110.9996,313.02938	+H	ESI+
Pesticide	Carboxin	5234-68-4	C12H13NO2S	235.0667	236.0736	0.85	7.25	150.56	93.0573,218.06341,166.03211,190.03211	+H, +Na	ESI+
Pesticide	Fenobucarb	3766-81-2	C12H17NO2	207.12593	230.1153	-1.59	1.79	150.26	152.0707,95.0491	+Na	ESI+
Pesticide	Lenacil	2164-08-1	C13H18N2O2	234.13683	235.1440	2.01	6.74	156.06	136.0393,110.06004,80.04948	+H, +Na	ESI+
Pesticide	Triazophos	24017-47-8	C12H16N3O3PS	313.065	314.0721	1.28	10.76	168.92	119.06037,286.04097,258.00967,96.95076	+H, +Na	ESI+
Pesticide	Mecarbam	2595-54-2	C10H20NO5PS2	329.05205	352.0417	-0.15	6.2	169.31	226.9957,144.0653	+Na	ESI+
Pesticide	Ethion	563-12-2	C9H22O4P2S4	383.9876	384.9949	1.56	14.26	175.22	96.95076,170.96978	+H	ESI+
Pesticide	Dichlofenthion	97-17-6	C10H13Cl2O3PS	313.97001	314.9770	0.92	11.62	158.58	286.94598,258.9147	+H	ESI+
Pesticide	Tebufenozide	112410-23-8	C22H28N2O2	352.21508	375.2045	-0.79	11.51	197.95	297.15975,105.06988	+Na	ESI+
Pesticide	Fluquinconazole	136426-54-5	C16H8Cl2FN5O	375.00899	376.0164	1.89	10.19	178.61	306.98357,272.01472,331.97882,163.0302	+H	ESI+
Pesticide	Chinomethionate	2439-01-2	C10H6N2OS2	233.9921	234.9994	2.56	11.76	145	173.0168	+H	ESI+
Pesticide	Benzyladenine	1214-39-7	C12H11N5	225.10145	226.1086	2.00	3.91	152.5	148.06177,184.08692	+H, +Na	ESI+
Pesticide	Pymetrozin	123312-89-0	C10H11N5O	217.09636	218.1035	2.03	1.68	155.44	78.03383,79.04165,105.0447	+H, +Na	ESI+
Pesticide	Pyrazoxyfen	71561-11-0	C20H16Cl2N2O3	402.0538	425.0427	-1.99	11.41	193.33	139.0502,172.95555,249.04253,285.01921	+Na, +H	ESI+
Pesticide	Saflufenacil	372137-35-4	C17H17ClF4N4O5S	500.0544	523.0432	-1.86	9.75	211.96	197.97526,366.02631,459.01476	+Na, +H	ESI+

Pesticide	Flutriafol	76674-21-0	C16H13F2N3O	301.10267	302.1097	1.10	7.74	167.84	95.02915,214.05886,215.06668,70.03997	+H	ESI+
Pesticide	Methoxyfenozide	161050-58-4	C22H28N2O3	368.20999	391.1993	-1.06	10.59	302.29	219.14919,313.15467	+Na, +H	ESI+
Pesticide	Flonicamid	158062-67-0	C9H6F3N3O	229.0463	230.0535	2.18	3.41	148.81	148.03686,176.03177,174.01612,183.0364	+H	ESI+
Pesticide	Fluridone	59756-60-4	C19H14F3NO	329.10275	330.1104	2.89	9.21	175.3	259.09917,290.09757,294.0725	+H, +Na	ESI+
Pesticide	Pyraflufen-ethyl	129630-17-7	C13H9Cl2F3N2O4	383.98915	384.9969	2.73	11.95	188.98	260.99922,253.01746,288.99414,304.0221	+H	ESI+
Pesticide	Trichlorfon	52-68-6	C4H8Cl3O4P	255.9225	256.9299	2.74	3.99	138.49	109.00491,78.9934	+H	ESI+
Pesticide	Chromafenozide	143807-66-3	C24H30N2O3	394.22564	417.2150	-0.86	10.92	207.36	189.10224,72.08078	+Na, +H	ESI+
Pesticide	Oxabetrinil	74782-23-3	C12H12N2O3	232.0847	233.0921	3.02	7.04	156.46	104.0468	+H	ESI+
Pesticide	Pyridalyl	179101-81-6	C18H14Cl4F3NO3	490.965	491.9710	-1.43	17.13	199.46	204.06308,183.02073,146.02121,148.0368	+H, +Na	ESI+
Pesticide	Cloquintocet-mexyl	99607-70-2	C18H22ClNO3	335.12882	358.1183	-0.66	12.89	180	238.02655,179.01324	+Na, +H	ESI+
Pesticide	Acrinathrin	101007-06-1	C26H21F6NO5	541.13239	542.1394	0.57	14.7	213.05	498.14602	+H	ESI+
Pesticide	Sulfentrazone	122836-35-5	C11H10Cl2F2N4O3<br>S	385.98187	408.9708	-1.99	7.52	172.32	273.03492,279.98505,366.98292,286.9897	+Na, +H	ESI+
Pesticide	Furathiocarb	65907-30-4	C18H26N2O5S	382.15624	405.1459	-0.10	13.52	188.37	149.05971,164.08318,147.04406,133.0647	+Na, +H	ESI+
Pesticide	Fluroxypyr	69377-81-7	C7H5Cl2FN2O3	253.96613	254.9732	1.46	5.98	139.42	208.96792,196.96792	+H	ESI+
Pesticide	Teflubenzuron	83121-18-0	C14H6Cl2F4N2O2	379.97425	380.9815	1.45	12.69	170	158.0411	+H	ESI+
Pesticide	Norflurazon	27314-13-2	C12H9ClF3N3O	303.03862	304.0460	2.24	8.24	158	264.03344,160.03686,140.03063,145.0259	+H, +Na	ESI+
Pesticide	Bensulide	741-58-2	C14H24NO4PS3	397.0605	398.0678	1.51	11.95	195.12	141.00048,218.0304,313.97388,77.03858	+H	ESI+
Pesticide	Napropamide	15299-99-7	C17H21NO2	271.15723	294.1466	-1.22	10.38	167.68	128.06205,127.05423,199.07536	+Na, +H	ESI+
Pesticide	Isoxathion	18854-01-8	C13H16NO4PS	313.05377	336.0432	-0.86	12.61	175.26	162.05495,239.98788,77.03858,286.02974	+Na, +H	ESI+
Pesticide	Fluopicolide	239110-15-7	C14H8Cl3F3N2O	381.96543	382.9730	2.28	10.49	172.95	108.98395,172.9614	+H, +Na	ESI+
Pesticide	Fluometuron	2164-17-2	C10H11F3N2O	232.08235	233.0896	2.37	7.45	145.81	160.03686,145.02596,140.03063,188.0317	+H, +Na	ESI+
Pesticide	Rimsulfuron	122931-48-0	C14H17N5O7S2	431.05694	454.0454	-2.88	7.39	189.59	139.0502,156.07675,233.98893	+Na, +H	ESI+
Pesticide	Fluopyram	658066-35-4	C16H11ClF6N2O	396.04641	397.0540	2.25	10.59	173.12	173.02088,145.02596,172.03686	+H, +Na	ESI+
Pesticide	Tribromsalan	87-10-5	C13H8Br3NO2	446.8105	445.8032	3.13	14.07	170.62	248.8556	-H	ESI-

Pesticide	Fluazuron	86811-58-7	C20H10Cl2F5N3O3	505.00194	506.0092	1.11	13.77	203.26	141.01465,158.0412	+H, +Na	ESI+
Pesticide	Novaluron	116714-46-6	C17H9ClF8N2O4	492.0123	493.0191	0.20	13.05	192.22	158.0412	+H	ESI+
Pesticide	Tebuconazol	107534-96-3	C16H22ClN3O	307.14514	308.1528	3.13	10.52	164.79	125.01525,151.0309	+H	ESI+
Pesticide	Isoxaflutole	141112-29-0	C15H12F3NO4S	359.04391	360.0512	1.64	9.74	180	144.01814,143.01031	+H	ESI+
Pesticide	Thidiazuron	51707-55-2	C9H8N4OS	220.0418	221.0492	3.18	6.05	144.76	127.99131,102.012	+H	ESI+
Pesticide	Procymidone	32809-16-8	C13H11Cl2NO2	283.01668	284.0243	3.25	10.86	163	256.0291	+H	ESI+
Pesticide	Fenpropimorph	67564-91-4	C20H33NO	303.2562	304.2630	0.33	9.18	172.83	119.08553,117.06988,132.09335,130.1226	+H	ESI+
Pesticide	Triallate	2303-17-5	C10H16Cl3NOS	303.00182	304.0084	-0.40	14.34	159.08	82.94498,261.96214	+H	ESI+
Pesticide	Prosulfuron	94125-34-5	C15H16F3N5O4S	419.08751	420.0947	1.17	9.57	182.16	141.07709,237.01916	+H, +Na	ESI+
Pesticide	Fenthion	55-38-9	C10H15O3PS2	278.02002	279.0270	1.01	11.71	154.06	169.0132	+H	ESI+
Pesticide	Simazine	122-34-9	C7H12ClN5	201.07812	202.0855	3.38	6.15	141.82	132.0323,124.08692,68.02432,166.10872	+H	ESI+
Pesticide	Flumiclorac-pentyl	87546-18-7	C21H23ClFNO5	423.12488	446.1151	1.23	13.81	204.53	354.0539,174.01165	+Na, +H	ESI+
Pesticide	Tebuthiuron	34014-18-1	C9H16N4OS	228.10448	229.1116	1.84	6.01	148.86	116.02769,157.06682,142.04334,156.0589	+H, +Na	ESI+
Pesticide	Lufenuron	103055-07-8	C17H8Cl2F8N2O3	509.97842	510.9851	-0.04	13.65	193.13	158.0412,327.97251	+H	ESI+
Pesticide	Pirimicarb	23103-98-2	C11H18N4O2	238.14298	239.1501	1.76	4.35	154.13	150.10257,167.10531,72.04439	+H, +Na	ESI+
Pesticide	Diphenylamine	122-39-4	C12H11N	169.08915	170.0963	2.66	10.46	137.07	92.04948	+H	ESI+
Pesticide	Fluconazole	86386-73-4	C13H12F2N6O	306.10407	307.1112	1.40	4.18	163.44	238.07864,169.04595,151.03538,127.0353	+H	ESI+
Pesticide	Flunixin meglumine	42461-84-7	C21H28F3N3O7	491.1879	492.1947	0.20	8.32	159.74	264.0505,259.06775,210.07876,239.06152	+H	ESI+
Pesticide	Cyclanilide	113136-77-9	C11H9Cl2NO3	272.99595	274.0026	-0.18	9.49	155.8	159.97153	+H	ESI+
Pesticide	Cyflufenamide	180409-60-3	C20H17F5N2O2	412.12102	413.1284	1.65	12.97	178.74	221.03325,295.08643k241.03948,183.0164	+H, +Na	ESI+
Pesticide	Clothianidin	210880-92-5	C6H8ClN5O2S	249.0087	250.0161	2.81	4.03	147.01	110.07127,167.03859	+H	ESI+
Pesticide	Spirodiclofen	148477-71-8	C21H24Cl2O4	410.10516	433.0943	-1.37	15.3	207.19	71.0859,295.02871	+Na, +H	ESI+
Pesticide	Propaquizafop	111479-05-1	C22H22ClN3O5	443.1248	444.1318	0.68	13.21	195.03	327.0531,371.07931,255.03197	+H, +Na	ESI+
Pesticide	Quinoxyfen	124495-18-7	C15H8Cl2FNO	306.9967	308.0041	2.28	12.62	159.67	213.9821,196.97936,287.99775	+H	ESI+
Pesticide	Sulfosulfuron	141776-32-1	C16H18N6O7S2	470.06784	493.0569	-1.36	8.38	198.17	261.02882,272.99982,156.07675,182.0560	+Na, +H	ESI+

									2		
Pesticide	Pyraclostrobin	175013-18-0	C19H18ClN3O4	387.09858	410.0877	-1.50	12.17	180.76	324.05343,261.08966,356.07965,218.0811	+Na, +H	ESI+
Pesticide	Ethoxysulfuron	126801-58-9	C15H18N4O7S	398.08962	421.0786	-1.81	9.82	187.04	182.05602,154.0611,139.03763	+Na, +H	ESI+
Pesticide	Thiacloprid	111988-49-9	C10H9ClN4S	252.02364	253.0307	1.43	5.49	153.28	90.03383,226.02002,99.00115,217.05424	+H, +Na	ESI+
Pesticide	Primisulfuron-methyl	86209-51-0	C15H12F4N4O7S	468.03628	491.0249	-2.31	10.3	194.08	204.02152,199.00596,186.01096,135.0440	+Na, +H	ESI+
Pesticide	Imazamox	114311-32-9	C15H19N3O4	305.13756	306.1451	2.75	4.65	168.69	261.12337,193.06077,264.09788,163.0502	+H, +Na	ESI+
Pesticide	Epoxiconazole	133855-98-8	C17H13ClFN3O	329.07312	330.0804	1.76	10.07	169.68	95.02915,74.0151	+H, +Na	ESI+
Pesticide	Fenhexamid	126833-17-8	C14H17Cl2NO2	301.06363	302.0711	2.56	10.24	162.73	142.00542,177.9821,97.10118	+H	ESI+
Pesticide	Clodinafop-propargyl	105512-06-9	C17H13ClFNO4	349.05171	350.0588	1.12	11.9	176.65	222.01165,238.00656	+H, +Na	ESI+
Pesticide	Cyprodinil	121552-61-2	C14H15N3	225.1266	226.1341	3.55	8.49	150.8	184.08692,93.0573,118.05255	+H	ESI+
Pesticide	Quinclorac	84087-01-4	C10H5Cl2NO2	240.96973	241.9765	0.29	6.01	138.83	195.97153,194.96371	+H	ESI+
Pesticide	Tribenuron-methyl	101200-48-0	C15H17N5O6S	395.0899	396.0969	0.76	8.55	185.65	181.072,199.00596,135.04406	+H	ESI+
Pesticide	Fenbuconazole	114369-43-6	C19H17ClN4	336.1141	337.1215	2.08	10.72	178.86	128.06205.89.03858	+H	ESI+
Pesticide	Isoprothiolane	50512-35-1	C12H18O4S2	290.0646	291.0716	1.03	10.5	160.34	144.97763,130.9256	+H	ESI+
Pesticide	Flumetsulam	98967-40-9	C12H9F2N5O2S	325.0445	326.0518	1.85	4.9	161.75	109.03223,128.03063,191.99253	+H, +Na	ESI+
Pesticide	Hexythiazox	78587-05-0	C17H21ClN2O2S	352.10123	353.1088	2.47	13.96	181.09	228.02444,115.05423,194.03672,151.0309	+H, +Na	ESI+
Pesticide	Bensulfuron-methyl	83055-99-6	C16H18N4O7S	410.08962	433.0789	-1.02	8.64	190.58	149.05971,119.04914,139.0502,91.05423	+Na, +H	ESI+
Pesticide	Triasulfuron	82097-50-5	C14H16ClN5O5S	401.05607	402.0634	1.57	7.3	181.89	141.07709	+H, +Na	ESI+
Pesticide	Fluazinam	79622-59-6	C13H4Cl2F6N4O4	463.95138	464.9587	1.34	13.8	181.23	376.96504	+H	ESI+
Pesticide	Flutolanil	66332-96-5	C17H16F3NO2	323.11331	346.1023	-2.20	10.79	169.38	262.06741,304.11436,145.02596,282.0736	+Na, +H	ESI+
Pesticide	Imazapyr	81334-34-1	C13H15N3O3	261.11134	262.1185	1.76	3.74	153.65	217.09715,199.08659,220.07167,149.0345	+H, +Na	ESI+
Pesticide	Clofentezine	74115-24-5	C14H8Cl2N4	302.0126	303.0199	1.99	12.11	159.86	138.0107,156.0213	+H, +Na	ESI+
Pesticide	Thiodicarb	59669-26-0	C10H18N4O4S3	354.04902	355.0563	1.64	7.24	186.04	88.0216,107.9937	+H, +Na	ESI+
Pesticide	Cymoxanil	57966-95-7	C7H10N4O3	198.0752	199.0826	3.53	3.72	137.53	111.0189	+H	ESI+
Pesticide	Haloxyfop	69806-34-4	C15H11ClF3NO4	361.03287	362.0403	2.02	10.93	176.02	288.00337,272.00845	+H	ESI+

Pesticide	Penconazole	66246-88-6	C13H15Cl2N3	283.0643	284.0712	0.71	9.15	161.26	172.99193.70.03997.137.01525	+H, +Na	ESI+
Pesticide	Pyrazophos	13457-18-6	C14H20N3O5PS	373.08613	374.0940	3.14	11.97	186.6	222.08732,176.04545,346.0621	+H, +Na	ESI+
Pesticide	Resmethrin	10453-86-8	C22H26O3	338.18819	339.1954	1.51	15.65	182.16	171.08044	+H, +Na	ESI+
Pesticide	Quizalofop Ethyl	76578-14-8	C19H17ClN2O4	372.08768	373.0951	1.94	12.94	188.03	255.03197,163.00575,207.08899	+H, +Na	ESI+
Pesticide	Oxyfluorfen	42874-03-3	C15H11ClF3NO4	361.03287	362.0403	2.02	13.73	171.3	297.98772,273.99016	+H	ESI+
Pesticide	Chlorsulfuron	64902-72-3	C12H12ClN5O4S	357.02985	358.0369	0.98	7.42	171.52	141.07709	+H, +Na	ESI+
Pesticide	Ethephon	16672-87-0	C2H6ClO3P	143.9743	144.9816	4.17	0.8	127.16	109.0048	+H	ESI+
Pesticide	Oxathiapiprolin	1003318-67-9	C24H22F5N5O2S	539.14144	562.1301	-1.93	10.94	205.21	522.13815,350.11332,480.13003	+Na, +H	ESI+
Pesticide	DEF 6	78-48-8	C12H27OPS3	314.09616	315.1037	2.67	15.21	173.58	112.92792,259.04084	+H, +Na	ESI+
Pesticide	Thiobencarb	28249-77-6	C12H16ClNOS	257.06411	258.0709	0.35	12.17	153.87	125.015,89.0386	+H	ESI+
Pesticide	Hexazinone	51235-04-2	C12H20N4O2	252.15863	253.1657	1.47	6.12	157.91	171.0867	+H	ESI+
Pesticide	Fenamiphos	22224-92-6	C13H22NO3PS	303.1058	326.0952	-0.99	10.09	167.77	201.9848,234.03483,184.98206,276.08178	+Na, +H	ESI+
Pesticide	Ethametsulfuron-methyl	97780-06-8	C15H18N6O6S	410.10085	411.1082	1.58	7.61	192.44	196.0829,142.07234,83.02399	+H, +Na	ESI+
Pesticide	Mesosulfuron-methyl	208465-21-8	C17H21N5O9S2	503.07807	504.0851	0.66	7.96	195.21	306.01005,139.0502,162.05495	+H, +Na	ESI+
Pesticide	Imazapic	104098-48-8	C14H17N3O3	275.12699	276.1345	2.94	4.63	159.34	163.0502,231.1128,234.08732,213.10224	+H, +Na	ESI+
Pesticide	Diafenthiuron	80060-09-9	C23H32N2OS	384.22353	385.2308	1.48	15.02	199.8	236.10699,287.12126	+H, +Na	ESI+
Pesticide	Triadimefon	43121-43-3	C14H16ClN3O2	293.0931	294.1002	1.36	10.15	168.65	126.99452,110.9996	+H	ESI+
Pesticide	Tricyclazole	41814-78-2	C9H7N3S	189.03607	190.0435	3.86	5.04	132.93	146.0059	+H, +Na	ESI+
Pesticide	Sumithrin	26002-80-2	C23H26O3	350.1881	351.1955	2.00	16.19	186.56	156.0936,143.0858,129.0698	+H	ESI+
Pesticide	Propanil	709-98-8	C9H9Cl2NO	217.0061	218.0135	3.23	8.85	127.01	160.97936	+H	ESI+
Pesticide	Picloram	1918-02-1	C6H3Cl3N2O2	239.926	240.9328	0.42	3.45	136.08	159.95896,222.92272	+H	ESI+
Pesticide	Iprodione	36734-19-7	C13H13Cl2N3O3	329.0334	330.0404	0.91	10.84	175.15	271.12412,231.09282,229.07717	+H	ESI+
Pesticide	Trimethoprim	738-70-5	C14H18N4O3	290.13789	291.1453	2.45	3.29	175.93	335.04299,291.10157,189.05462	+H, +Na	ESI+
Pesticide	Brodifacoum	56073-10-0	C31H23O3Br	522.08306	523.0897	-0.11	15.39	224.1	238.09749,258.10372,252.11314	+H, +Na	ESI+
Pesticide	Isopyrazam	881685-58-1	C20H23F2N3O	359.18092	360.1882	1.61	12.42	186.55	250.0987,244.0886	+H, +Na	ESI+

Pesticide	Cyproconazole	94361-06-5	C15H18ClN3O	291.11384	292.1205	-0.14	9.42	170.28	125.0143,70.0403	+H	ESI+
Pesticide	Fenamidone	161326-34-7	C17H17N3OS	311.10923	312.1165	1.83	9.96	173.75	165.0481,264.11314,295.08996,239.11789	+H, +Na	ESI+
Pesticide	Mandestrobin	173662-97-0	C19H23NO3	313.1677	314.1751	2.24	11.05	180.27	189.08966	+H	ESI+
Pesticide	Molinate	2212-67-1	C9H17NOS	187.10308	188.1104	3.31	9.56	137.69	126.0913,83.0855	+H	ESI+
Pesticide	Cyclaniliprole	1031756-98-5	C21H17Br2Cl2N5O2	598.91261	621.9009	-2.35	11.23	217.89	177.00883,205.00374,514.83073,257.8952	+Na, +H	ESI+
Pesticide	Aclonifen	74070-46-5	C12H9ClN2O3	264.03017	265.0371	0.87	11.15	156.6	182.0605,218.0363	+H	ESI+
Pesticide	Benalaxyl	71626-11-4	C20H23NO3	325.16779	348.1571	-1.20	11.99	173.77	65.038,77.0379	+Na, +H	ESI+
Pesticide	Dimethachlor	50563-36-5	C13H18ClNO2	255.1026	256.1099	2.35	8.48	155.54	224.0837	+H	ESI+
Pesticide	halauxifen-methyl	943831-98-9	C14H11Cl2FN2O3	344.01308	345.0202	1.22	9.82	170.4	235.0069,253.98083,312.99414	+H, +Na	ESI+
Pesticide	Thiophanate-methyl	23564-05-8	C12H14N4O4S2	342.04565	343.0531	2.19	6.67	175.84	311.0263,151.0312	+H	ESI+
Pesticide	Chlorotoluron	15545-48-9	C10H13ClN2O	212.07164	213.0788	2.17	7.41	144.91	168.02107,125.01525	+H, +Na	ESI+
Pesticide	Valifenalate	283159-90-0	C19H27ClN2O5	398.16085	421.1506	0.13	9.96	199.36		+Na, +H	ESI+
Pesticide	Clopidol	2971-90-6	C7H7Cl2NO	190.99047	191.9977	2.78	2.46	131.08	157.02889,176.97427	+H, +Na	ESI+
Pesticide	Myclobutanil	88671-89-0	C15H17ClN4	288.1141	289.1215	2.43	9.96	170	164.02615,125.0142	+H, +Na	ESI+
Pesticide	Tau-Fluvalinate	102851-06-9	C26H22ClF3N2O3	502.1271	503.1347	1.79	16.36	207.36	208.07569,250.06049	+H	ESI+
Pesticide	Flamprop-methyl	52756-25-9	C17H15ClFNO3	335.07245	336.0797	1.64	10.55	178.71	304.05351,105.03349	+H	ESI+
Pesticide	Maleic Hydrazide	123-33-1	C4H4N2O2	112.0272	113.0344	4.46	0.78	120		+H	ESI+
Pesticide	Metolachlor	51218-45-2	C15H22ClNO2	283.13391	306.1233	-1.09	10.69	167.74	106.06513,103.05423,176.10699,202.1226	+Na, +H	ESI+
Pesticide	Bromacil	314-40-9	C9H13BrN2O2	260.016	261.0233	2.31	6.06	123.19	187.93417,161.9549	+H	ESI+
Pesticide	Allethrin	584-79-2	C19H26O3	302.1881	303.1955	2.32	13.7	161.28	135.0795,151.1106	+H	ESI+
Pesticide	Spiroxamine	118134-30-8	C18H35NO2	297.26678	298.2741	2.09	9.21	178.73	100.11208,72.08078,160.13321,254.24784	+H	ESI+
Pesticide	Monolinuron	1746-81-2	C9H11ClN2O2	214.05091	215.0581	2.29	7.55	141.95	98.999,127.0177	+H	ESI+
Pesticide	Carbosulfan	55285-14-8	C20H32N2O3S	380.21336	381.2206	1.42	16.65	200.77	165.09101,123.04406,118.0685	+H	ESI+
Pesticide	Bioresmethrin	28434-01-7	C22H26O3	338.18819	339.1955	1.80	15.62	183.14	247.13287,171.08044,159.08044,173.0961	+H, +Na	ESI+

Pesticide	Buprofezin	69327-76-0	C16H23N3OS	305.15618	306.1636	2.36	10.9	171.69	201.1057,116.0529	+H, +Na	ESI+
Pesticide	Boscalid	188425-85-6	C18H12Cl2N2O	342.03267	343.0399	1.55	10.12	168.87	111.99485.139.98977,76.01818	+H	ESI+
Pesticide	Coumaphos	56-72-4	C14H16ClO5PS	362.01446	363.0219	2.04	12.08	175.41	288.94857,306.95913,211.01565,334.9904	+H, +Na	ESI+
Pesticide	Diazinon	333-41-5	C12H21N2O3PS	304.10105	305.1083	1.81	11.97	169.95	96.95076,84.04439,231.03516,277.07703	+H	ESI+
Pesticide	Warfarin	81-81-2	C19H16O4	308.10486	331.0945	-0.19	9.19	171.69	163.03897,173.02332,121.02841,291.1015	+Na, +H	ESI+
Pesticide	Metribuzin	21087-64-9	C8H14N4OS	214.08883	215.0961	2.66	6.29	145.85	186.08214	+H	ESI+
Pesticide	Alachlor	15972-60-8	C14H20ClNO2	269.1182	270.1255	2.23	10.65	151.9	162.1269,103.05423	+H	ESI+
Pesticide	Aminopyralid	150114-71-9	C6H4Cl2N2O2	205.96498	206.9722	2.52	1.67	132.01	125.99793,188.96169	+H	ESI+
Pesticide	Benalaxyl-M	98243-83-5	C20H23NO3	325.1677	326.1730	-4.31	11.52	161.74	208.13321,133.0886,91.05423,121.0886	+H	ESI+
Pesticide	Flazasulfuron	104040-78-0	C13H12F3N5O5S	407.05112	430.0401	-1.77	8.66	182.19	139.0502,227.00966,146.02121,156.07675	+Na, +H	ESI+
Pesticide	Atrazine	1912-24-9	C8H14ClN5	215.09377	216.1010	2.46	7.55	150.44	146.0228,132.0323,104.001,68.02432	+H	ESI+
Pesticide	Fluoroimide	41205-21-4	C10H4Cl2FNO2	258.9603	259.9676	2.32	5.41	149.72		+H	ESI+
Pesticide	Cyantraniliprole	736994-63-1	C19H14BrClN6O2	472.00501	494.9926	-4.47	8.05	200.11	441.97009,177.00883,185.03455,111.9948	+Na, +H	ESI+
Pesticide	Prometryn	7287-19-6	C10H19N5S	241.1361	242.1434	2.49	7.78	162.02	200.09644,68.02432,116.02769,85.05087	+H	ESI+
Pesticide	Propazine	139-40-2	C9H16ClN5	229.10942	230.1167	2.53	8.86	156.49	146.0228,110.04612,104.001,68.02432	+H	ESI+
Pesticide	MGK 264	113-48-4	C17H25NO2	275.18853	276.1958	2.07	12.83	169.82	98.02365	+H	ESI+
Pesticide	S-metolachlor	87392-12-9	C15H22ClNO2	283.13391	284.1412	2.08	10.69	167.74	224.08367,105.06988,107.08553,89.03858	+Na, +H	ESI+
Pesticide	Ametoctradin	865318-97-4	C15H25N5	275.211	276.2185	2.91	9.84	173.81	149.08217,190.10872,162.07742,164.0931	+H, +Na	ESI+
Pesticide	Tolfenpyrad	129558-76-5	C21H22ClN3O2	383.14005	384.1471	0.91	13.24	204.59	171.03197145.0527,182.07262,117.0214	+H, +Na	ESI+
Pesticide	Indaziflam	950782-86-2	C16H20FN5	301.17027	302.1776	2.09	8.86	175.98	158.08365,145.10118,130.0777,105.06988	+H, +Na	ESI+
Pesticide	Vinclozoline	50471-44-8	C12H9Cl2NO3	284.9959	286.0032	2.11	9.8	157.02	214.0184	+H	ESI+
Pesticide	Tetrachlorvinphos	961-11-5	C10H9O4PCl4	363.89926	386.8882	-2.09	10.85	170.52	238.89834,168.96063,167.95281,202.9216	+Na, +H	ESI+
Pesticide	Diallate	2303-16-4	C10H17NOSCl2	269.0407	270.0476	0.74	13.07	156.56	108.96063,142.94835	+H	ESI+
Pesticide	EPTC	759-94-4	C9H19NOS	189.1187	190.1260	3.17	11.02	142.99		+H	ESI+
Pesticide	Imazaquin	81335-37-7	C17H17N3O3	311.1269	312.1343	2.25	6.44	171.07	252.07675,267.1128,198.06619,181.03964	+H	ESI+

Pesticide	Tridemorph	24602-86-6	C19H39NO	297.30316	298.3107	2.83	11.08	188.01	112.11208	+H	ESI+
Pesticide	Thiophanate	23564-06-9	C14H18N4O4S2	370.07695	371.0842	1.49	8.57	185.4	160.05054,118.05255,93.0573,235.99468	+H, +Na	ESI+
Pesticide	Enilconazole	35554-44-0	C14H14Cl2N2O	296.0483	297.0556	2.03	7.44	166.07	255.00864,172.99193,200.98685,137.0152	+H	ESI+
Pesticide	Pindone	83-26-1	C14H14O3	230.09429	231.1012	0.91	12.38	147.51		+H	ESI+
Pesticide	Chloroxuron	1982-47-4	C15H15ClN2O2	290.08221	291.0894	1.69	9.73	173.17	183.06787,72.04439,211.06278,245.02381	+H, +Na	ESI+
Pesticide	Etrimfos	38260-54-7	C10H17N2O4PS	292.06466	293.0719	1.85	11.68	162.27	233.01443,124.98206,109.00325,142.9926	+H, +Na	ESI+
Pesticide	Cyanophos	2636-26-2	C9H10NO3PS	243.0119	244.0192	2.47	9	149.87	124.9821,230.0039	+H	ESI+
Pesticide	Nicosulfuron	111991-09-4	C15H18N6O6S	410.10085	433.0896	-2.32	6.16	191.49	139.0502,213.03284,106.02874,156.07675	+Na, +H	ESI+
Pesticide	Clethodim	99129-21-2	C17H26ClNO3S	359.1321	360.1399	3.06	13.22	189.2	136.07569,206.11756,240.10528,136.0756	+H, +Na	ESI+
Pesticide	Pyridaphenthion	119-12-0	C14H17N2O4PS	340.06466	341.0719	1.59	10.24	175.03	313.04064,171.05529,285.00934,172.0393	+H, +Na	ESI+
Pesticide	Guazatine	115044-19-4	C18H41N7	355.3423	356.3501	3.10	1.93	185.31	170.16517,153.13863,187.19172,195.1604	+H	ESI+
Pesticide	Bioallethrin	28057-48-9	C19H26O3	302.1881	303.1959	3.64	13.71	173.17	121.06479	+H	ESI+
Pesticide	Triflumuron	64628-44-0	C15H10ClF3N2O3	358.0332	359.0406	1.96	11.68	170.36	156.02107,110.9996,178.04742,203.01886	+H, +Na	ESI+
Pesticide	Pyraclofos	77458-01-6	C14H18ClN2O3PS	360.04643	361.0535	1.03	11.85	180.87	333.0224,272.96489,290.97545,319.00675	+H, +Na	ESI+
Pesticide	Tetraconazole	112281-77-3	C13H11Cl2F4N3O	371.02153	372.0294	3.15	10.42	174.58	333.0224,272.96489,290.97545,319.00675	+H	ESI+
Pesticide	Fenpyroximate	111812-58-9	C24H27N3O4	421.20016	422.2073	1.04	14.27	216.9	214.09749,138.06619,231.10023,202.0974	+H	ESI+
Pesticide	Bifenazate	149877-41-8	C17H20N2O3	300.14739	301.1547	2.03	10.5	180.41	184.07569,259.10772,166.06513,244.0842	+H	ESI+
Pesticide	Trifloxystrobin	141517-21-7	C20H19N2O4F3	408.12969	409.1370	1.49	13.08	188.29	145.02596,206.08117,146.06004.143.0365	+H	ESI+
Pesticide	Carfentrazone-ethyl	128639-02-1	C15H14Cl2F3N3O3	411.03643	412.0437	1.39	11.45	181.5	366.00184,240.99365,276.97033,302.0302	+H	ESI+
Pesticide	Indoxacarb	144171-61-9	C22H17ClF3N3O7	527.07071	550.0593	-2.11	12.99	201.5	249.04253,190.00542,217.01632,219.0319	+Na, +H	ESI+
Pesticide	Bonzi	76738-62-0	C15H20N3OCl	293.12949	294.1369	2.42	9.19	166.54	70.03997,89.03858,139.0309,207.0935	+H	ESI+
Pesticide	Cinosulfuron	94593-91-6	C15H19N5O7S	413.10052	414.1076	0.92	6.94	186.14	183.01104,157.072,83.02399,141.00048	+H, +Na	ESI+
Pesticide	2.4.6-Trichlorophenol	88-06-2	C6H3Cl3O	195.9249	194.9177	-2.55	25.06	142.56	158.9412	-H	ESI-
Pesticide	Difenoconazole	119446-68-3	C19H17Cl2N3O3	405.0647	406.0723	2.22	11.81	195.1	323.02363,202.01799,261.99467,309.0079	+H, +Na	ESI+
Pesticide	Metamitron	41394-05-2	C10H10N4O	202.08546	203.0928	3.17	3.96	140.38	174.07876,145.03964,77.03858,186.06619	+H	ESI+

Pesticide	Picoxystrobin	117428-22-5	C18H16F3NO4	367.10314	390.0926	-0.65	11.76	173.98	115.05423,205.08592,177.09101	+Na	ESI+
Pesticide	Flubendiamide	272451-65-7	C23H22F7IN2O4S	682.02332	705.0127	-0.47	11.71	230.17	407.9761,358.06611,135.04743,606.98061	+Na	ESI+
Pesticide	Tolclofos-methyl	57018-04-9	C9H11Cl2O3PS	299.9543	300.9616	2.00	12.29	152.51	174.9712,253.91194,218.94309,146.97628	+H	ESI+
Pesticide	Trinexapac-ethyl	95266-40-3	C13H16O5	252.09977	253.1070	2.10	8.31	150.69	165.01824,207.06519,137.02332,139.0389	+H	ESI+
Pesticide	Spirotetramat	203313-25-1	C21H27NO5	373.18892	396.1784	-0.59	9.89	183.17	302.17507,270.14886,244.13321,188.1069	+Na, +H	ESI+
Pesticide	Halosulfuron methyl	100784-20-1	C13H15ClN6O7S	434.04115	457.0300	-1.96	9.68	191.44	139.0502,403.02221,221.97347,247.95273	+Na, +H	ESI+
Pesticide	Mandipropamid	374726-62-2	C23H22ClNO4	411.12374	434.1131	-0.83	10.28	190.71	125.01525,164.07061,395.09189,289.0864	+Na, +H	ESI+
Pesticide	Amidosulfuron	120923-37-7	C9H15N5O7S2	369.04129	370.0484	1.11	7.21	170.19	139.03763,154.0611,182.05602,124.05054	+H, +Na	ESI+
Pesticide	Pretilachlor	51218-49-6	C17H26ClNO2	311.16521	334.1548	-0.35	12.72	175.98	176.14338,132.08078,147.10425,117.0573	+Na, +H	ESI+
Pesticide	Fipronil sulfide	120067-83-6	C12H4Cl2F6N4S	419.94379	420.9513	1.93	12.45	173.15	289.97611,350.94803,212.94802	+H	ESI+
Pesticide	Haloxyfop-ethoxyethyl	87237-48-7	C19H19ClF3NO5	433.0903	434.0977	1.62	13.39	181.85	288.0422,272.00845,344.02958,388.0558	+H	ESI+
Pesticide	Fenthion-sulfone	3761-42-0	C10H15O5PS2	310.00985	311.0172	2.10	8.32	162.18	200.00554,246.9647,168.98715,233.03958	+H, +Na	ESI+
Pesticide	Imibenconazole	86598-92-7	C17H13Cl3N4S	409.9926	410.9999	1.46	12.94	180.8	341.96723,181.98257,251.01527,306.9983	+H	ESI+
Pesticide	Thifluzamide	130000-40-7	C13H6Br2F6N2O2S	525.84209	526.8493	0.97	11.56	177.73	168.00893	+H	ESI+
Pesticide	Mepronil	55814-41-0	C17H19NO2	269.1415	270.1487	1.86	10.42	157.38	91.05423,119.04914,136.0393,210.09134	+H	ESI+
Pesticide	Simetryn	1014-70-6	C8H15N5S	213.10482	214.1122	3.19	5.51	146.28	68.02432,124.08692,144.05899,166.10872	+H	ESI+
Pesticide	Mesotrione	104206-82-8	C14H13NO7S	339.0412	340.0485	1.77	6.46	165.23	104.01309,293.04782,170.00322	+H	ESI+
Pesticide	Imidaclothiz	105843-36-5	C7H8ClN5O2S	261.00872	283.9973	-4.29	4.53	156.65	122.07127,180.04642,123.0791,154.04334	+Na, +H	ESI+
Pesticide	Fenoxanil	115852-48-7	C15H18Cl2N2O2	328.0745	329.0818	1.83	11.48	162.4	141.11482,126.09134	+H	ESI+
Pesticide	Fluthiacet-methyl	117337-19-6	C15H15ClFN3O3S2	403.02274	404.0306	2.88	11.22	182.3	331.00106,273.99736,214.98405,187.9731	+H, +Na	ESI+
Pesticide	Cyazofamid	120116-88-3	C13H13ClN4O2S	324.0447	325.0521	2.16	11.53	167	181.06345	+H, +Na	ESI+
Pesticide	Prothioconazole-desthio	120983-64-4	C14H15Cl2N3O	311.05922	312.0661	0.58	9.71	161.42	153.06988,154.0777,70.03997,152.06205	+H	ESI+
Pesticide	Ethoprophos	13194-48-4	C8H19O2PS2	242.0564	243.0637	2.48	10.09	141.46	130.93848,172.98543,78.9402	+H	ESI+

Pesticide	Pyriftalid	135186-78-6	C15H14N2O4S	318.06743	319.0749	2.42	9.59	173.39	273.06922,179.01613,287.04849,241.0430	+H, +Na	ESI+
Pesticide	Cyclosulfamuron	136849-15-5	C17H19N5O6S	421.1056	422.1129	1.42	10.29	198.06	182.05602,139.03763,154.0611,156.07675	+H, +Na	ESI+
Pesticide	Florasulam	145701-23-1	C12H8F3N5O3S	359.02999	360.0372	1.42	6.74	165.32	109.03223,191.99253,128.03063,108.0244	+H, +Na	ESI+
Pesticide	8-Hydroxyquinoline	148-24-3	C9H7NO	145.05276	146.0601	4.41	1.88	128.73	118.065	+H	ESI+
Pesticide	Nitenpyram	150824-47-8	C11H15ClN4O2	270.0884	271.0956	1.85	3.2	147.23	169.0527,196.0636,188.11822,190.13387	+H	ESI+
Pesticide	Oxaziclomefone	153197-14-9	C20H19Cl2NO2	375.0792	376.0866	1.87	13.58	179.82	161.05971,133.06479,115.05423,187.0075	+H	ESI+
Pesticide	Zoxamide	156052-68-5	C14H16Cl3NO2	335.02466	336.0320	1.91	11.83	172.38	158.97628,203.99775,122.9996,124.00743	+H, +Na	ESI+
Pesticide	Alar	1596-84-5	C6H12N2O3	160.0847	161.0921	4.37	3.19	116.07	143.0802	+H	ESI+
Pesticide	Dimethenamide-P	163515-14-8	C12H18ClNO2S	275.07468	298.0636	-2.84	9.51	162.71		+Na, +H	ESI+
Pesticide	Pyribenzoxim	168088-61-7	C32H27N5O8	609.18596	632.1754	-0.43	13.87	259.72	180.08078,381.08296,300.0615,275.06625	+Na, +H	ESI+
Pesticide	Edifenphos	17109-49-8	C14H15O2PS2	310.02511	333.0140	-2.61	11.19	163.92	109.01065,154.9715,172.98206,77.03858	+Na, +H	ESI+
Pesticide	Prothioconazole	178928-70-6	C14H15Cl2N3OS	343.0312	344.0386	2.04	10.74	168.61	154.0777,189.04655	+H	ESI+
Pesticide	Ethiprole	181587-01-9	C13H9Cl2F3N4OS	395.9826	396.9899	1.52	9.5	173.8	368.9586,240.95416,283.94914,367.95077	+H	ESI+
Pesticide	Kasugamycin	6980-18-3	C14H25N3O9	379.159	380.1654	-0.79	0.51	185	112.0753,156.1087	+H	ESI+
Pesticide	Oxadiazon	19666-30-9	C15H18Cl2N2O3	344.06945	345.0772	3.05	4.45	179.1	297.0821,303.0294	+H, +Na	ESI+
Pesticide	Fipronil Desulfinyl	205650-65-3	C12H4Cl2F6N4	387.97172	388.9789	1.24	11.9	168.15	306.99302,334.00392,348.96654,212.9480	+H	ESI+
Pesticide	Flumorph	211867-47-9	C21H22FNO4	371.15329	394.1426	-1.24	8.45	196.33	165.05462,257.09723,242.07376,227.0502	+Na, +H	ESI+
Pesticide	Orthosulfamuron	213464-77-8	C16H20N6O6S	424.1165	447.1053	-2.29	7.82	192.04	182.05602,156.07675,199.08257,120.0443	+Na, +H	ESI+
Pesticide	Penoxsulam	219714-96-2	C16H14F5N5O5S	483.06358	484.0712	1.90	8.49	189.22	195.07508,194.06725,164.05669,166.0723	+H, +Na	ESI+
Pesticide	Phosalone	2310-17-0	C12H15ClNO4PS2	366.9868	367.9941	1.63	12.44	172.49	138.0105	+H, +Na	ESI+
Pesticide	Enoxastrobin	238410-11-2	C22H22ClNO4	399.12374	422.1129	-1.35	13.64	198.79	178.0418,102.0464,115.05423,145.06479	+Na	ESI+
Pesticide	Ethirimol	23947-60-6	C11H19N3O	209.1528	210.1601	2.87	4.46	132.82	140.10699,165.10224,98.06004,166.09749	+H	ESI+
Pesticide	Pinoxaden	243973-20-8	C23H32N2O4	400.23621	401.2436	1.72	11.32	191.17	289.15467,244.12063,288.14684,271.1441	+H, +Na	ESI+
Pesticide	Metamifop	256412-89-2	C23H18ClFN2O4	440.09391	463.0827	-2.07	12.88	196.08	180.08192,162.09134,244.01598,161.0835	+Na, +H	ESI+
Pesticide	Iprobenfos	26087-47-8	C13H21O3PS	288.0949	311.0840	-2.08	10.74	164.8		+Na	ESI+

Pesticide	1,2-Benzisothiazol-3(2H)-one	2634-33-5	C7H5NOS	151.00918	152.0166	4.77	3.66	124.03	134.0047	+H	ESI+
Pesticide	Oxydemeton-methyl	301-12-2	C6H15O4PS2	246.01494	269.0041	-2.19	2.96	148.02	124.98206,109.00491	+Na, +H	ESI+
Pesticide	Fenamiphos sulfoxide	31972-43-7	C13H22NO4PS	319.10072	342.0901	-1.00	6.34	170.58	292.07669,250.02974,277.05322,216.0005	+Na, +H	ESI+
Pesticide	Fenamiphos sulfone	31972-44-8	C13H22NO5PS	335.09563	358.0850	-0.98	7.18	173.23	188.04711,308.07161,248.99811,218.9875	+Na, +H	ESI+
Pesticide	N-2,4-Dimethylphenyl-N'-methylformamidine	33089-74-6	C10H14N2	162.1157	163.1230	3.70	3.48	131.52	117.0573,107.07295,132.08078,122.09643	+H	ESI+
Pesticide	Butralin	33629-47-9	C14H21N3O4	295.1532	296.1605	2.03	14.39	159.13	223.09514，4,2.098	+H	ESI+
Pesticide	Isoproturon	34123-59-6	C12H18N2O	206.14191	207.1492	2.86	7.86	155.4	165.1011	+H	ESI+
Pesticide	Oxadiargyl	39807-15-3	C15H14Cl2N2O3	340.03815	341.0456	2.21	12.35	174.31	239.96136,213.9821,257.97192,217.94062	+H	ESI+
Pesticide	Flucetosulfuron	412928-75-7	C18H22FN5O8S	487.11731	488.1247	1.42	8.61	199.15		+H, +Na	ESI+
Pesticide	Isazophos	42509-80-8	C9H17ClN3O3PS	313.04168	336.0309	-1.53	11.18	170.04	135.97424,215.9394,243.9707,96.95076	+Na, +H	ESI+
Pesticide	Niclosamide	50-65-7	C13H8Cl2N2O4	325.9861	326.9934	1.84	11.42	158.33	174.9921,251.0001	+H	ESI+
Pesticide	Oxycarboxin	5259-88-1	C12H13NO4S	267.05653	290.0452	-3.86	5.69	162.44	160.07569	+Na	ESI+
Pesticide	Metalaxyl	57837-19-1	C15H21NO4	279.14706	302.1362	-2.01	8.06	159.26	145.0886,130.06513,220.13321,148.11208	+Na, +H	ESI+
Pesticide	Chlordimeform	6164-98-3	C10H13ClN2	196.07673	197.0840	2.91	3.91	143.6	125.01525,152.02615	+H	ESI+
Pesticide	3,5-Dichloroaniline	626-43-7	C6H5NCl2	160.9799	161.9872	3.73	8.54	130,8	144.96063	+H	ESI+
Pesticide	Fenothiocarb	62850-32-2	C13H19NO2S	253.1136	254.1193	-3.95	10.79	157		+H	ESI+
Pesticide	Anilofos	64249-01-0	C13H19ClNO3PS2	367.0232	368.0306	1.91	11.84	169.56	170.96978,166.00542,198.9647,156.95413	+H	ESI+
Pesticide	forchlorfenuron	68157-60-8	C12H10ClN3O	247.0512	248.0585	2.43	7.43	147.73	155.00067,93.04472,119.02399,91.02907	+H	ESI+
Pesticide	Fluazifop	69335-91-7	C15H12F3NO4	327.07184	328.0793	2.32	9.52	169.82	254.04234	+H	ESI+
Pesticide	Haloxyfop-methyl	69806-40-2	C16H13ClF3NO4	375.04852	376.0558	1.55	12.71	181.39	288.0422,272.00845	+H, +Na	ESI+
Pesticide	Fluazifop-butyl	69806-50-4	C19H20F3NO4	383.1344	384.1417	1.57	13.85	189.93	328.07912,254.08117,238.04742	+H, +Na	ESI+
Pesticide	Mefenacet	73250-68-7	C16H14N2O2S	298.0775	299.0849	2.35	9.95	163.21	136.02155,192.01138,77.03858	+H, +Na	ESI+
Pesticide	Metsulfuron-methyl	74223-64-6	C14H15N5O6S	381.0743	382.0819	2.36	6.94	180.98	199.00596,135.04406,209.98555,263.9947	+H, +Na	ESI+

Pesticide	Oxadixyl	77732-09-3	C14H18N2O4	278.12666	279.1339	1.94	6.33	160.21	132.079,117.055	+H, +Na	ESI+
Pesticide	Imazethapyr	81335-77-5	C15H19N3O3	289.1426	290.1499	2.08	5.64	165.02	230.0924,245.12845,248.10297,227.11789	+H	ESI+
Pesticide	Clomazon	81777-89-1	C12H14ClNO2	239.0713	240.0786	2.51	8.68	146.73	125.0147,89.03858	+H	ESI+
Pesticide	Ametryn	834-12-8	C9H17N5S	227.12047	228.1280	3.65	6.65	153.5	158.04949,68.02432,138.07742,144.05899	+H	ESI+
Pesticide	Uniconazole	83657-22-1	C15H18N3OCl	291.1138	292.1211	2.06	9.98	168.56	218.04795,70.03997,170.109,125.01525	+H	ESI+
Pesticide	Diniconazol	83657-24-3	C15H17Cl2N3O	325.0748	326.0821	1.85	11.07	173.91	70.03997,308.07158,252.00898,204.07003	+H	ESI+
Pesticide	Rotenone	83-79-4	C23H22O6	394.14164	395.1491	1.93	10.97	189.9	203.07027,177.05462,198.06753,367.154	+H, +Na	ESI+
Pesticide	Flusilazole	85509-19-9	C16H15F2N3Si	315.10033	316.1076	1.81	10.58	170.01	219.04361,227.06868,151.03738,217.0279	+H	ESI+
Pesticide	Hexaflumuron	86479-06-3	C16H8Cl2F6N2O3	459.9816	460.9889	1.28	12.52	189.04	158.0412	+H	ESI+
Pesticide	Propisochlor	86763-47-5	C15H22ClNO2	283.13391	306.1229	-2.51	11.67	170.49	184.99031,121.02706,143.00067,160.0272	+Na	ESI+
Pesticide	Chlorimuron-ethyl	90982-32-4	C15H15ClN4O6S	414.04008	437.0294	-0.92	9.76	189.65		+Na, +H	ESI+
Pesticide	Ethoxyquin	91-53-2	C14H19NO	217.14666	218.1541	3.41	7.57	152.01	160.07569,188.10699,190.12264,146.0600	+H	ESI+
Pesticide	Pyriproxyfen	95737-68-1	C20H19NO3	321.13649	322.1438	1.90	13.59	174.82	227.10666,78.03383,199.07536,96.04439	+H	ESI+
Pesticide	Fosthiazate	98886-44-3	C9H18NO3PS2	283.04657	284.0538	1.87	7.61	162.15	227.9907,104.0163	+H	ESI+
Pesticide	Sulcotrione	99105-77-8	C14H13ClO5S	328.01722	329.0244	1.46	6.89	166.9	139.03897,249.0313,68.99711,111.04406	+H, +Na	ESI+
Pesticide	Spinetoram	187166-40-1	C42H69NO10	747.4921	748.4999	1.47	12.52	275.08	98.09643,629.36841	+H	ESI+
Pesticide	Fenthion sulfoxide	3761-41-9	C10H15O4PS2	294.01494	295.0221	1.56	7.21	162.79	261.98817,264.00382,168.98715,232.0317	+H, +Na	ESI+
Pesticide	Flufiprole	704886-18-0	C16H10Cl2F6N4OS	489.98566	490.9922	-0.33	12.78	183.97	421.99772,387.02887,408.9899,359.00726	+H, +Na	ESI+
Pesticide	Aspoxicillin	63358-49-6	C21H27N5O7S	493.16312	494.1699	0.16	2.35	215.84	250.11862,357.11416,476.15983,349.0852	+H, +Na	ESI+
Pesticide	Deltamethrin	52918-63-5	C22H19Br2NO3	504.9711	505.9777	-0.20	15.71	202.6	217.08592,488.96955,424.05428,91.05423	+H, +Na	ESI+
Pesticide	Cypermethrin	52315-07-8	C22H19Cl2NO3	415.0741	416.0817	2.17	17.29	202.68	191.0026,182.0558	+H	ESI+
Pesticide	Methomyl	16752-77-5	C5H10N2O2S	162.0462	163.0534	3.09	9.52	132.48	163.0534	+H	ESI+
Pesticide	Carbofuran	1563-66-2	C12H15NO3	221.1051	222.1125	3.17	7.01	151.94	165.09101,145.01583,147.08044,137.0597	+H	ESI+
Pesticide	3-Hydroxycarbofuran	16655-82-6	C12H15NO4	237.1001	238.1074	2.53	4.37	152.42	135.08044,145.06479,161.05971,208.0730	+H	ESI+

Pesticide	Carbaryl	63-25-2	C12H11NO2	201.07898	224.0678	-4.38	6.5	147.11		+Na	ESI+
Pesticide	Carbendazim	10605-21-7	C9H9N3O2	191.0694	192.0767	3.14	2.53	139.07	133.06345,90.03383,92.04948,159.04271	+H	ESI+
Pesticide	Imidacloprid	138261-41-3	C9H10ClN5O2	255.0523	256.0593	1.18	4.33	156.48	175.09782	+H	ESI+
Pesticide	Phoxim	14816-18-3	C12H15N2O3PS	298.0541	299.0611	1.01	12.53	167.6	96.95076,224.98821	+H	ESI+
Pesticide	Acetamiprid	135410-20-7	C24H38N4O5	222.06722	223.0743	1.71	4.77	153.52	90.03383,187.09782,181.0527	+H, +Na	ESI+
Pesticide	Pyridaben	96489-71-3	C19H25ClN2OS	364.1376	365.1449	1.65	15.19	192.11	79.05423	+H	ESI+
Pesticide	Pyrimethanil	53112-28-0	C12H13N3	199.1109	200.1182	3.01	6.64	145.97	107.06037,119.06037,170.07127,80.04948	+H	ESI+
Pesticide	Dimethomorph	110488-70-5	C21H22NO4Cl	387.12374	388.1312	1.96	9.53	202.81	165.05462,273.06768,258.04421	+H, +Na	ESI+
Pesticide	Prochloraz	67747-09-5	C15H16Cl3N3O2	375.0308	376.0381	1.60	9.79	185.86	244.02905,265.95369,201.9821,222.94787	+H	ESI+
Pesticide	Azoxystrobin	131860-33-8	C22H17N3O5	403.1168	404.1241	1.49	10.02	199.69	169.03964,343.07133,190.06245,253.0607	+H	ESI+
Pesticide	Thiamethoxam	153719-23-4	C8H10ClN5O3S	291.0192	292.0266	2.41	3.53	161.05	211.06481,152.02769,180.04642	+H	ESI+
Pesticide	Abamectin	65195-55-3	C48H72O14	872.4922	873.4995	0.69	15.29	305.72	855.48892,305.21112,567.33163,449.2533	+H	ESI+
Pesticide	Diflubenzuron	35367-38-5	C14H9ClF2N2O2	310.032	311.0391	1.29	10.5	165.04	158.0412,113.01973,114.02756	+H	ESI+
Pesticide	Chlorobenzuron	57160-47-1	C14H10Cl2N2O2	308.01193	309.0193	2.18	10.86	164.31	156.02107,110.9996	+H	ESI+
Pesticide	Dichlorvos	62-73-7	C4H7Cl2O4P	219.9459	220.9531	2.27	6.41	135.73	94.94498	+H	ESI+
Pesticide	Metamidophos	10265-92-6	C2H8NO2PS	141.0013	142.0086	4.26	1.33	130.93	94.0052,124.9824	+H	ESI+
Pesticide	Malathion	121-75-5	C10H19O6PS2	330.036	331.0433	1.82	7.92	185.4	99.00767	+H	ESI+
Pesticide	Phosphamidon	13171-21-6	C10H19NO5PCl	299.0689	300.0769	4.35	6.26	159.83	174.06802,109.00491,226.98706	+H	ESI+
Pesticide	Parathion	56-38-2	C10H14NO5PS	291.033	292.0403	2.06	3.17	157.66	246.0474	+H	ESI+
Pesticide	Omethoate	1113-02-6	C5H12NO4PS	213.0224	214.0297	2.82	2.05	137.89	154.99263,109.00491,164.9644,150.94875	+H	ESI+
Pesticide	Dimethoate	60-51-5	C5H12NO3PS2	228.9996	230.0069	2.62	8.98	140.22	153.00079,167.01644,135.99805,151.9929	+H	ESI+
Pesticide	Monocrotophos	6923-22-4	C7H14NO5P	223.0609	224.0682	2.69	3.17	148.88	148.99982	+H	ESI+
Pesticide	Dursban	2921-88-2	C9H11Cl3NO3PS	348.9262	349.9336	2.01	13.99	166.51	293.87096,275.8604,161.9508,96.95076	+H	ESI+
Pesticide	Methyl parathion	298-00-0	C8H10NO5PS	263.0017	264.0090	2.28	9.85	152.73	249.9938	+H	ESI+
Pesticide	Chlorpyrifos-methyl	5598-13-0	C7H7Cl3NO3PS	320.8949	321.9020	1.25	13.99	155.92	293.87096,275.8604,161.9508,96.95076	+H	ESI+

Pesticide	Fenitrothion	122-14-5	C9H12NO5PS	277.0173	278.0241	0.36	7.97	161.27	245.99844,217.00828,230.02129,232.0317	+H	ESI+
Pesticide	Pirimiphos-methyl	29232-93-7	C11H20N3O3PS	305.0962	306.1036	2.29	11.67	168.32	164.11822,274.07736,246.04606,108.0556	+H	ESI+
Pesticide	Pirimiphos-ethyl	23505-41-1	C13H24N3O3PS	333.1275	334.1349	2.10	13.38	180.44	306.10358,154.09749,260.06171,278.0722	+H	ESI+
Pesticide	Profenofos	41198-08-7	C11H15BrClO3PS	371.9351	372.9431	3.50	12.82	169.89	284.8536,128.00234,205.93527	+H	ESI+
Pesticide	Propachlor	1918-16-7	C11H14ClNO	211.0763	212.0837	3.32	8.45	145.7	94.06513	+H	ESI+
Pesticide	Cyanazine	21725-46-2	C9H13ClN6	240.089	241.0961	1.67	6.39	158.53	205.11962,174.0541,173.05885,178.10872	+H	ESI+
Pesticide	Dinitramine	29091-05-2	C11H13F3N4O4	322.0888	323.0962	2.17	11.94	162.31	289.09069,247.04374,261.05939	+H	ESI+
Pesticide	Benodanil	15310-01-7	C13H10INO	322.9807	323.9878	1.24	8.69	150.7	202.93522,76.03075	+H	ESI+
Pesticide	Chlorbufam	101-21-3	C10H12ClNO2	213.0556	214.0629	2.82	9.4	144.38	110.9996	+H	ESI+
Pesticide	N,N'-Diphenylethanediamide	620-81-5	C14H12N2O2	240.0898	241.0967	0.83	8.78	156.39	162.09134,146.06004	+H	ESI+
Pesticide	Bromuconazol	116255-48-2	C13H12BrCl2N3O	374.954	375.9614	1.87	9.7	171.11	227.0025,306.92866,208.99193,70.03997	+H	ESI+
Pesticide	(E)-Fenpyroximate	134098-61-6	C24H27N3O4	421.2001	422.2074	1.42	14.29	221.77	214.09749,138.06619,231.10023,202.09749	+H	ESI+
Pesticide	Fenpropidin	67306-00-7	C19H31N	273.2456	274.2529	2.20	8.56	170.1	117.06988,119.08553,132.09335	+H	ESI+
Pesticide	Benoxacor	98730-04-2	C11H11Cl2NO2	259.0166	260.0236	1.16	9.4	150.51	149.08352,188.07061,120.04439,196.0523	+H	ESI+
Pesticide	Etoxazole	153233-91-1	C21H23F2NO2	359.1696	360.1770	1.95	14.37	190	304.11436,177.12739,113.01973	+H	ESI+
Pesticide	Butafenacil	134605-64-4	C20H18ClF3N2O6	474.0805	475.0867	-1.05	11.42	215.21	179.98468,349.01975	+H	ESI+
Pesticide	Butamifos	36335-67-8	C13H21N2O4PS	332.09596	331.0904	3.43	12.95	171.85	152.0353,295.9440,394.93621,275.02609	-H	ESI-
Pesticide	Isofenphos	25311-71-1	C15H24NO4PS	345.11637	344.1094	-0.78	12.99	173.87	256.02027,137.02442	-H	ESI-
Pesticide	Asulam	3337-71-1	C8H10N2O4S	230.03613	229.0284	-4.48	1.49	141.23	197.00264	-H	ESI-
Pesticide	Fomesafen	72178-02-0	C15H10ClF3N2O6S	437.99002	436.9833	-0.05	8.59	188.39	194.983,222.01967,315.99938,285.99574	-H	ESI-
Pesticide	Cyhalofop	122008-78-0	C16H12FNO4	301.07504	300.0672	-3.79	5.89	231.81	208.0404,228.04663,120.0255,180.04281	-H	ESI-
Pesticide	Sulfoxaflor	946578-00-3	C10H10F3N3OS	277.04967	276.0421	-3.14	5.75	157.63	212.04251	-H	ESI-

Pesticide	Ioxynil	1689-83-4	C7H3I2NO	370.8304	369.8223	-3.78	6.35	137.03	126.90502,242.91866	-H	ESI-
Pesticide	Dinoterb	1420-07-1	C10H12N2O5	240.07462	239.0677	-0.92	8.37	145.07	207.04113,176.03532,208.04896	-H	ESI-
Pesticide	Bentazon	25057-89-0	C10H12N2O3S	240.05686	239.0495	-2.75	3.43	141.63	197.0026,132.0329,175.08769,133.04074	-H	ESI-
Pesticide	Bromoxynil	1689-84-5	C7H3Br2NO	274.85814	273.8507	-2.69	4.57	143.59	78.91889,168.92945,193.9247	-H	ESI-
Toxin	α-Zearalanol	26538-44-3	C18H26O5	322.178	323.1853	1.86	8.29	158.3	305.1747,167.0339,163.07536,189.05462	+H	ESI+
Toxin	Aflatoxin M1	6795-23-9	C17H12O7	328.0582	329.0651	0.61	5.36	164.75	273.07563,229.04951,259.05988,301.0705	+H	ESI+
Toxin	Aflatoxin M2	6885-57-0	C17H14O7	330.0739	331.0821	4.54	4.94	166.49	273.0757,259.0600,285.0758,313.07058	+H	ESI+
Toxin	Aflatoxin G1	1165-39-5	C17H12O7	328.0582	329.0662	3.96	6.08	163.85	243.0650,311.0549,283.059,268.0357	+H	ESI+
Toxin	Aflatoxin G2	7241-98-7	C17H14O7	330.0739	331.0809	0.91	5.65	165.55	313.0706,285.0757,245.0808,189.0546	+H	ESI+
Toxin	Aflatoxin B1	1162-65-8	C17H12O6	312.0633	313.0703	0.96	6.58	161.55	285.0753,241.0493,269.0443,270.05106	+H	ESI+
Toxin	Aflatoxin B2	7220-81-7	C17H14O6	314.079	315.0854	-0.96	6.15	163	287.0910,259.059,243.0651,271.06013	+H	ESI+
Toxin	Alternariol	641-38-3	C14H10O5	258.0528	259.0596	0.39	6.68	152.01	213.0544,185.0596,128.0618	+H	ESI+
Toxin	Tenuazonic acid	610-88-8	C10H15NO3	197.1051	198.1117	-0.51	6.13	142.53	153.091,181.0858	+H	ESI+
Toxin	Tentoxin	28540-82-1	C22H30N4O4	414.2267	415.2338	0.97	7.62	202.4	302.1497,199.1437,312.1703	+H	ESI+
Toxin	Altenuene	889101-41-1	C15H16O6	292.0946	293.1017	1.37	5.84	165.18	275.0913,279.0932,280.097	+H	ESI+
Toxin	3-acetyldeoxynivalenol	50722-38-8	C17H22O7	338.13657	339.1433	0.07	3.93	168.35	305.139,28287.12923	+H	ESI+
Toxin	Citrinin	518-75-2	C13H14O5	250.08412	251.0909	0.11	7.46	151.76	233.08011,205.08545,215.069,191.06968	+H	ESI+
Toxin	Deoxynivalenol	51481-10-8	C15H20O6	296.12598	297.1328	0.28	2.55	162.69	203.10647	+H	ESI+
Toxin	Fumonisin B1	116355-83-0	C34H59NO15	721.3884	722.3952	0.01	7.35	254.8	704.3844,334.3103,352.3209,686.37409	+H	ESI+
Toxin	Fumonisin B2	116355-84-1	C34H59NO14	705.3935	706.4003	0.06	8.72	256.36	336.32627,688.3901,318.3157,512.3581	+H	ESI+
Toxin	Fumonisin B3	1422359-85-0	C34H59NO14	705.3935	706.4003	0.14	8.18	254.11	336.32625,688.3901,318.3157,354.33671	+H	ESI+
Toxin	HT-2 toxin	26934-87-2	C22H32O8	424.20972	425.2165	0.20	7.72	191.3	263.1275,245.1179,105.0703,347.18364	+H	ESI+
Toxin	Neosolaniol	36519-25-2	C19H26O8	382.1627	383.1701	1.83	6.72	181.23	367.17332,356.27999	+H	ESI+
Toxin	Resiniferatoxin	57444-62-9	C37H40O9	628.2672	629.2751	1.91	9.36	236.44	351.1563,293.1531,,311.1641,517.18324	+H	ESI+
Toxin	Sterigmatocystin	10048-13-2	C18H12O6	324.0633	325.0708	2.47	9.8	163.76	281.04429,310.04699,253.0494,254.0560	+H	ESI+

Toxin	T-2 toxin	21259-20-1	C24H34O9	466.2202	467.2271	0.27	9.21	198.85	263.1276,213.0939,423.16407,300.09982	+H	ESI+
Toxin	Wortmannin	19545-26-7	C23H24O8	428.1471	429.1537	-0.29	2.2	186.13	429.1548	+H	ESI+
Toxin	Zearalenone	36455-70-6	C18H22O5	318.1467	317.1401	0.31	9.45	172.62	301.14351,187.0756,185.0595,,203.07015	-H	ESI-

Table S2. Quantitative limit, recovery, and RSD of harmful substances in milk due to regulatory differences and MRL in different countries and regions

Compound	LOQ (μg /kg)	1 μg /kg		5μg /kg		10 μg /kg		MRL (μg /kg)				
		Recovery (%)	RSD (%)	Recovery (%)	RSD (%)	Recovery (%)	RSD (%)	China	European Union	New Zealand	Japan	USA
3-Hydroxycarbofuran	1	114.2	7.0	116.6	1.7	119.7	15.9	/	/	/	/	/
Ametoctradin	1	118.5	8.0	103.9	2.7	118.8	17	/	30	/	/	/
Ametryn	1	113.1	3.1	109.6	3.4	108.6	2.1	/	/	/	/	/
Anilofos	1	102.3	4.1	107.9	3.8	98.8	17.2	/	/	/	/	/
Atrazine	1	94.4	10.9	108.2	5.7	116.9	6.8	/	10	/	20	20
Azaperone	1	111.4	4.8	110.2	2.2	108.8	4	/	/	/	/	/
Benalaxyl	1	96.9	1.3	111.1	2.7	116.7	20.2	/	/	/	/	/
Bensulide	1	112.1	6.8	87.9	2.2	94.6	8.8	/	/	/	/	/
Benzyladenine	1	118.7	1.7	102.7	2.4	116.2	12.6	/	/	/	/	/
Bifenazate	1	70.8	10.3	81.2	4.3	85.4	10.7	100	20	/	10	/
Buprofezin	1	96.5	1.9	102.4	1.9	104.2	3	/	/	/	/	/
Carazolol	1	115	3.4	107	3.2	101.9	9.4	/	1	/	1	/
Carfentrazone-ethyl	1	116.3	5	110.3	3.4	113.6	16.1	/	/	/	40	/
Chlordimeform	1	91.7	2.3	101.1	2.6	108.5	2.9	/	/	/	/	/
Chromafenozide	1	73.5	17.4	69.7	10.5	74.6	6.8	/	10	/	/	/
Cloquintocet-mexyl	1	102.1	5.7	108.5	4.6	94.6	2.9	/	/	/	/	/

Cyanazine	1	96.2	2.6	107.9	3.1	91.9	5.3	/	/	/	/	/
Cyclaniliprole	1	80.5	6.5	77.3	8.4	88.5	4.5	/	10	/	/	/
Cyproconazole	1	91.4	5.4	91.7	1.9	90.7	7.1	/	50	10	10	/
Diazinon	1	115	3.4	113.1	1.9	108.5	4.9	/	/	/	/	/
Difenoconazole	1	71.2	12.6	101.3	9.1	97.4	6.4	70	5	10	20	/
Dimethachlor	1	74	4.6	111.2	6.4	115.8	14.6	/	/	/	/	/
Diniconazol	1	79.4	4.6	103.1	3.1	91.1	5.4	/	10	/	/	/
Doramectin	1	80.9	7.4	113.3	4	92.8	4.8	15	/	15	15	/
Edifenphos	1	75.3	6.1	107.4	5.1	119.6	17.7	/	/	/	/	/
Enilconazole	1	85.4	2.7	107.8	4	98.6	6.8	/	/	/	/	/
Enoxacin	1	92.5	6.3	89.5	7.2	94.3	5.3	/	/	/	/	/
Enoxastrobin	1	116.3	3.6	105.5	4.4	119.1	15.7	/	/	/	/	/
Ethirimol	1	111.4	2.6	97.1	2.7	93.3	8.8	/	/	/	/	/
Etoxazole	1	119.2	2.3	105.4	3.8	102.5	5.6	/	10	/	10	10
Etrimfos	1	114.8	3.2	107.3	1.8	108.4	4.6	/	/	/	/	/
Fenamidone	1	91.9	3	110.4	1.9	106.3	8.4	100	10	/	20	/
Fenamiphos	1	106.3	5.4	109.8	2.1	93.6	11.9	700	5	10	5	/
Fenarimol	1	117.8	4	103.6	2.6	109.2	5.1	/	20	/	10	/
Fenpropidin	1	114.5	4.9	111	3.8	117.2	3.3	/	20	5	/	/
Fenpropimorph	1	69.3	15.4	66.5	8.6	75.4	8.3	30	15	10	10	/
Fenpyroximate	1	112.5	5.4	109.8	4.6	110.7	7.8	/	10	/	10	15
Fipronil Desulfinyl	1	76.3	6.4	81.5	6.1	88.9	5.6	20	/	/	/	/
Fluazifop	1	109.6	6.5	104.5	4.1	115.6	6	/	/	/	/	/
Fluazifop-butyl	1	115.2	3.8	98.3	3.5	91.1	4.8	/	/	/	/	/
Fluazinam	1	81.5	4.6	95.3	5.5	95.1	4.7	/	10	/	10	/

Fluazuron	1	91.2	12.6	97.5	10.1	93.1	4.5	/	/	/	/	/
Flubendazole	1	110.8	3	110.3	4.4	104.1	8.4	/	/	/	/	/
Flubendiamide	1	93.6	2.5	95.5	3.6	94.9	3.5	/	/	/	/	/
Fluconazole	1	82.3	18.7	105.8	2.7	98.5	5.8	/	/	/	/	/
Flufiprole	1	96.1	4.7	104.6	3.6	105.3	3.8	/	/	/	/	/
Flumorph	1	114.5	5.4	94.3	3.6	116.6	18	/	/	/	/	/
Fluopyram	1	113.9	6.5	107.7	1.6	106.9	16.3	20	/	300	600	/
Fluridone	1	77.7	4.5	111.7	1.4	108.5	3.3	/	/	/	/	/
Flusilazole	1	113.5	2.9	117.8	2.4	107.3	6.5	20	20	10	50	/
Fluthiacet-methyl	1	115.4	4.5	112.5	4.4	90.6	2.9	/	/	/	/	/
Flutriafol	1	115.9	4.9	110.1	5.3	114.6	5.1	10	10	/	50	20
Fosthiazate	1	78.8	2.2	82.1	5.1	99.6	17	/	/	/	/	/
Furathiocarb	1	94.6	1.7	113	3	103.9	3.7	/	/	/	/	/
Gamithromycin	1	111.6	5.4	94	4.1	95	11.1	/	/	/	10	/
Gatifloxacin	1	96.4	14.3	115.9	13.8	107.8	15.8	/	/	/	/	/
Halosulfuron methyl	1	75.2	5.3	77.9	4.8	80.2	4.8	/	10	/	/	/
Hexaflumuron	1	93.3	10.6	98.9	13.6	98.3	10.7	/	/	/	/	/
Hexazinone	1	89.2	1.6	107.5	3.7	90.8	18.4	/	/	/	11000	11000
Imazamox	1	89.6	3.2	70.1	3.6	76.2	11.3	/	/	/	/	/
Imazapic	1	63.8	9.4	84.1	13.4	68.2	3	/	/	/	/	/
Imazaquin	1	85.4	19.7	71.1	16.2	94.7	9.1	/	50	/	/	/
Imazethapyr	1	108.2	17.1	109.8	3.9	92.4	4.2	/	/	/	/	/
Indaziflam	1	115.9	1.4	110	2.9	103.9	9.6	/	/	/	/	/
Indoxacarb	1	94.4	4.5	109.2	5.1	99.2	15.5	/	100	/	100	4000
Iprobenfos	1	106.2	5.8	109.8	4.3	102.1	15.4	/	/	/	/	/

Isazophos	1	108.6	2.1	100.4	4.4	104.7	2.8	/	/	/	/	/
Isoprothiolane	1	84.4	1.6	110	3.6	91.7	13.3	/	/	/	/	/
Isoproturon	1	81.6	6.6	109.3	4.1	117.4	8.1	/	/	/	/	/
Isopyrazam	1	110.7	3.6	90.9	14.9	101.2	3.5	10	10	/	20	/
Isouron	1	118.5	3.8	106.9	4.4	98.4	6.3	/	/	/	/	/
Isoxaben	1	92.3	2.6	90.2	2	87.8	15	/	10	/	/	/
Isoxathion	1	118.7	3.6	108.8	5.8	101.5	10.8	/	/	/	/	/
Lufenuron	1	85.7	16.8	89.5	6.1	98.7	10	/	/	/	50	/
Mandestrobin	1	117.9	2.1	112.5	2.1	107.7	15	/	/	/	/	/
Mandipropamid	1	75.9	2.9	111.3	3.5	109.1	13.2	/	20	/	/	/
Mebendazole	1	85.2	2.5	104.4	3.5	114.4	7.5	/	/	/	/	/
Metamifop	1	110.8	3.4	105.8	2.9	116.5	13.5	/	/	/	/	/
Methoxyfenozide	1	101.5	4.2	109.8	5.2	113.5	6.3	/	50	/	50	100
Metoclopramide	1	114.2	16.5	85.3	9.4	104.3	3.5	/	/	/	/	/
Metribuzin	1	101.5	3.8	104.9	3.7	118.7	8.4	/	100	/	50	50
Monepantel	1	100.8	15.5	91.3	5.2	94.1	3.8	/	/	/	/	/
Myclobutanil	1	106.1	3.3	115.9	5.7	103.3	6.9	/	/	/	/	/
N-2,4-Dimethylphenyl-N'-methylformamidine	1	98.3	3.1	119.5	2.3	98.4	3	/	/	/	/	/
Napropamide	1	85.9	2.1	109.3	2.5	117.1	10.6	/	10	/	/	/
Niclosamide	1	89.3	6.3	90.5	3.9	93.1	3.9	/	/	/	/	/
Norflurazon	1	111.9	6.5	94.9	3.5	88.5	4.9	/	/	/	100	100
Oxathiapiprolin	1	93.8	5.1	118.5	4.4	98.1	3.6	10	10	/	/	/
Oxaziclomefone	1	113.3	3.2	108.2	1.7	92.9	6.4	/	/	/	/	/
Oxibendazole	1	108.1	8	108.9	4.5	117.4	11.5	/	/	/	/	/
Oxycarboxin	1	112.3	5.7	114.5	3.5	113.6	16.1	/	/	/	/	/

Phosphamidon	1	93.7	2.7	107.6	3.6	97.8	15.6	/	/	/	/	/
Picoxystrobin	1	86.4	2.8	109.9	2.6	94.3	7	/	10	/	10	/
Pinoxaden	1	94.3	6.3	89.8	5.8	105.9	5.6	/	10	/	20	/
Pirimicarb	1	109.5	3.6	111.9	2	101.6	3.1	/	/	/	50	/
Pirimiphos-ethyl	1	90.8	2.8	104.1	3.1	93.1	3.7	/	/	/	/	/
Pirimiphos-methyl	1	117	2.8	104.9	2	97.4	5	10	10	/	10	/
Pretilachlor	1	109.7	5.4	103.9	3.6	119.6	16.8	/	/	/	/	/
Prometryn	1	87.6	3.1	88.8	2.5	80.6	6.3	/	/	/	/	/
Propachlor	1	114.9	8.5	115.8	2.2	91.9	11.8	/	/	/	/	/
Propazine	1	118.5	10.9	105.3	2.1	113.6	7.2	/	/	/	/	/
Propyzamide	1	97.4	16.3	99.5	15.8	103.2	9.8	/	10	/	/	20
Prothioconazole	1	87.6	6.9	96.2	5.8	95.3	4.9	4	10	/	4	/
Pyraclostrobin	1	115.7	3.5	108.5	2.4	110.8	16.5	/	/	/	/	/
Pyrazophos	1	92.9	4.2	110.8	2.3	98.2	3.7	/	/	/	/	/
Pyrazoxyfen	1	104.2	1.5	107	3.4	94.2	6.8	/	/	/	/	/
Pyribenzoxim	1	113.1	3.3	109.8	4.7	90.6	8.2	/	/	/	/	/
Pyridaphenthion	1	104.9	14.3	105.5	3.9	110.4	17.2	/	/	/	/	/
Pyriftalid	1	70.7	3.5	110.2	2.8	92.7	10.8	/	/	/	/	/
Pyrimethanil	1	113.6	4.1	105.9	3.3	103.6	8.2	10	50	/	10	50
Pyriproxyfen	1	111.3	3.4	101.5	2.8	119.6	7.8	/	50	/	/	/
Quinoxyfen	1	108	2.2	95.6	4.6	106.9	12.8	20	50	/	10	/
Resiniferatoxin	1	89.2	7.9	115	3.2	101.1	15.5	/	/	/	/	/
Robenidine	1	116.7	3.4	99.8	2.8	98.4	7.7	/	/	/	10	/
Simazine	1	113	4.9	100.9	4.4	92.5	8.8	/	10	/	20	30
Simetryn	1	83.1	2.2	111	2.5	92.4	4.6	/	/	/	/	/

S-metolachlor	1	77.7	1.2	107.3	2.5	102.3	18.9	/	10	/	/	20
Spirotetramat	1	87.9	6.2	114.1	2.2	96.7	10.3	5	/	/	10	10
Spiroxamine	1	90.7	8.8	108.4	3.8	98.3	5.9	/	/	/	40	/
Sterigmatocystin	1	67.7	2.5	107.4	1.4	91.6	7.9	/	/	/	/	/
Sulfanitran	1	89.2	5.5	93.2	4.7	92.9	3.6	/	/	/	10	/
Sulfoxaflor	1	74.2	7.3	80.3	5.9	83.8	5.1	20	/	30	200	/
Tebuconazol	1	85.9	5.1	109.8	4.1	85.8	7.5	/	/	/	10	100
Tebufenozide	1	92.9	1.6	113.5	4.4	106.3	16.8	50	10	/	20	40
Tebuthiuron	1	115.4	4.2	104.3	2.4	107.3	6.8	/	/	/	/	/
Tetraconazole	1	115.7	3.1	102.5	2.4	99.2	4.1	/	/	/	/	/
Thiodicarb	1	107.2	2.3	101.6	3.6	110.5	5.9	/	10	/	20	/
Toltrazuril	1	103.2	4.2	105.6	3.7	103.8	4.6	/	/	/	/	/
Triazophos	1	80.9	4.1	113.4	3.3	99.6	16.1	/	10	/	/	/
Trimethoprim	1	101.1	6.7	93.1	4	93.5	11	50	50	/	50	/
Valifenalate	1	84.4	8.8	116.3	1.6	106.2	11.4	/	/	/	/	/
Zearalenone	1	110.5	3.6	113.6	6.8	114.8	8.1	/	/	/	/	/
Compound	LOQ (μg /kg)	5 μg /kg		10 μg /kg		25 μg /kg						
		Recovery (%)	RSD (%)	Recovery (%)	RSD (%)	Recovery (%)	RSD (%)					
Acrinathrin	5	65.7	10.5	70.6	10.5	70.3	6.8	/	10	/	/	/
Altrenogest	5	93.4	2.6	82.9	18.8	76.5	5.8	/	/	/	10	/
Azoxystrobin	5	112.2	4.1	102.3	17.3	101.5	1.9	800	10	/	10	6
Bensulfuron-methyl	5	102.3	10.7	104.3	11.2	114.7	7.4	/	10	/	/	/
Boscalid	5	102.6	14.8	119.3	4.8	103.1	5.1	10	20	100	100	100
Bromacil	5	89.1	11.3	88.5	13.6	81.6	5.6	/	/	/	/	/
Butamifos	5	73.9	15.6	86.5	5.8	89.5	3.8	/	/	/	/	/

Butralin	5	92.7	17	99.4	20	93.4	8.7	/	/	/	/	/
Chloridazon	5	113.4	6.4	119.7	13.9	99.1	4.1	/	/	/	/	/
Chlorimuron-ethyl	5	80.1	14.6	87.8	9.5	101.3	3.8	/	/	/	/	/
Chlorotoluron	5	92.1	17.9	91.7	9.4	95.1	2.5	/	/	/	/	/
Chloroxuron	5	109	7.8	95.7	4	102.9	3.4	/	20	/	/	/
Cinosulfuron	5	95.9	7.6	85.5	6.9	101.1	7.8	/	/	/	/	/
Cinoxacin	5	81	8.1	73.1	11.1	83.2	6.7	/	/	/	/	/
Ciprofloxacin	5	95.3	14.3	90.7	10.1	92.8	6.9	/	/	/	/	/
Clodinafop-propargyl	5	93.7	10.7	117.7	12.2	104.6	3.1	/	20	/	/	/
Clomazon	5	104.4	14.4	92.5	7	99	5.4	/	/	/	/	/
Coumaphos	5	106.3	6.2	113	4.3	108.1	6.1	/	/	/	/	/
Cyantraniliprole	5	105.3	16.3	107.6	13	113.2	6.3	/	20	10	600	200
Cyclanilide	5	94.6	7.9	99.1	5.3	96.2	5.3	/	/	/	/	/
Danofloxacin	5	83.6	18.4	94.1	10.7	89.5	9.9	30	30	/	50	/
Ceoxynivalenol	5	64.8	15.1	101	19.6	103.3	9	/	/	/	/	/
Dimethenamide-P	5	95.6	4.3	110.9	19.4	99.8	3.6	/	/	/	/	/
Dimethomorph	5	115.4	4.3	114	13.7	108.3	6	/	10	/	10	/
Dinoterb	5	92.5	6.3	98.6	6.2	103.6	5.5	/	10	/	/	/
Dursban	5	93.6	9.5	118.2	10.2	92	2.2	10	/	/	20	/
Enrofloxacin	5	83.6	13.6	89.3	8.2	90.3	6.1	100	100	/	50	/
Ethiprole	5	92.3	11	90.6	4.5	107.5	5.1	/	/	/	/	/
Ethoxysulfuron	5	113.4	9.4	101.8	11.6	97.4	3.5	/	/	/	/	/
Fenhexamid	5	82	13.7	82.1	11.1	112.3	1.4	/	10	/	10	/
Fenothiocarb	5	64.7	17.6	82.1	15.7	94.1	7.9	/	/	/	/	/
Flamprop-methyl	5	71.5	6	117.8	16	105.4	2.9	/	/	/	10	/

Flazasulfuron	5	113.5	8.2	117.4	7.2	96.6	5	/	/	/	/	/
Flucetosulfuron	5	100.4	6.3	93.6	10.9	99.4	5.8	/	/	/	/	/
Flumequine	5	102.3	8.7	111.1	8	97.4	2.8	50	50	/	50	/
Flumetsulam	5	101.7	5.2	90.4	3.7	101.1	4.8	/	/	/	100	/
Flumiclorac-pentyl	5	80.3	14.9	88.6	15.3	88.3	13	/	/	/	/	/
Flunixin meglumine	5	91.2	9	94.7	10.6	95.5	5.7	/	40	/	/	/
Fluquinconazole	5	86.5	9.3	86	7.1	95.7	6.2	/	30	/	100	/
Flutolanil	5	104.4	9.9	90.7	9.1	106.1	5.5	/	50	/	50	50
Fomesafen	5	106.3	6.4	109.5	4.1	110.5	5	/	10	/	/	/
Forchlorfenuron	5	110.3	7.2	116.2	4.9	88.2	2.3	/	10	/	/	/
Griseofulvin	5	112.1	4.6	108.5	13.7	113.8	4.1	/	/	/	/	/
Halauxifen-methyl	5	90.6	4.3	99.5	4.8	105.3	4.5	/	20	10	/	/
Haloxyfop	5	101.1	8.2	105.8	8.4	102.6	4.3	/	/	/	/	/
Haloxyfop-ethoxyethyl	5	112.4	13.5	105.6	6.3	101.6	3.5	/	/	/	/	/
Haloxyfop-methyl	5	104.5	5	91.1	5.9	102.2	4.1	/	/	/	/	/
Hexythiazox	5	87.5	15.7	86.2	6.4	87.9	7.2	50	50	/	50	50
Imazapyr	5	64.2	19.5	62.3	11.7	73.4	3.2	10	/	/	10	10
Imibenconazole	5	107.4	5.5	110.7	6.7	104.7	1.4	/	/	/	/	/
Ivermectin	5	85.1	10.7	95.3	6.3	97.6	5.8	10	/	10	10	/
Ketoprofen	5	78.5	14.1	77.4	9.5	86.4	7.6	/	/	/	30	/
Mefenacet	5	72.4	11.2	110.1	16.4	108.8	6.6	/	/	/	/	/
Mepronil	5	97.6	4.8	118.5	7	99.5	2.7	/	/	/	/	/
Metalaxyl	5	105.7	6.1	98.8	19	85.6	2.6	/	/	/	/	/
Metamitron	5	108.3	3.8	119	9.5	82.4	5.7	/	50	/	/	/
Metolachlor	5	89.7	5.9	92.6	5.5	95.5	7.4	/	10	/	/	20

Metosulam	5	108.9	7.4	107.6	4.3	98.1	6.6	/	10	/	10	/
Metsulfuron-methyl	5	107.4	17	110.9	14.8	88	5.1	/	10	/	70	50
Nalidixic acid	5	68.3	8.6	73.6	6.3	80.9	5.5	/	/	/	/	/
Nicosulfuron	5	68.8	16.4	99.2	19.3	90.6	6.7	/	20	/	/	10
Nitenpyram	5	74.5	15.8	75.8	13	86.2	4.1	/	/	/	/	/
Norfloxacin	5	89.3	17.9	77.8	18	93.5	10.2	/	/	/	10	/
Novaluron	5	106.4	12.1	107.5	12.1	113.8	2.9	/	400	/	400	1000
Ofloxacin	5	95.1	11.1	115.2	13.7	95.7	4.4	/	/	/	10	/
Omethoate	5	95.7	12	114.9	16	83.4	13.2	/	/	/	/	/
Oxolinic acid	5	115.3	6.3	106.4	7.2	114.8	6.4	/	/	/	10	/
Oxydemeton-methyl	5	89.9	3.5	84.1	6.4	119.7	2.1	/	/	/	/	/
Pefloxacin	5	75.6	10.8	79.6	12	85.1	5.6	/	/	/	/	/
Penconazole	5	77.8	5.7	83.2	4.9	85.6	4.3	10	10	/	10	/
Penoxsulam	5	115.6	5.5	108.4	12.2	100.9	2.6	/	/	/	10	/
Primisulfuron-methyl	5	89.3	9.6	93.8	6.8	118.3	10.4	/	/	/	/	/
Prochloraz	5	101.7	5.5	92.9	14.6	99.8	4.8	/	/	/	/	/
Profenofos	5	71	9	83.7	19.6	105.5	9.8	20	10	/	10	10
Propaquizafop	5	99.9	6.4	118.1	14.8	99.8	3.9	/	/	/	/	/
Propiconazole	5	84.9	6.4	84.3	2.9	100	4.8	10	10	/	10	50
Pyraclofos	5	107.2	11.7	96.1	4.7	103.1	4.8	/	/	/	/	/
Pyraflufen-ethyl	5	115.3	4.8	115.1	3.1	108.6	2.9	/	/	/	/	/
Pyridaben	5	90.9	13.5	103.2	11.7	82.4	2.8	/	10	/	10	10
Pyridalyl	5	61.8	2.9	62.6	3.5	75.8	1.3	/	10	/	/	/
Rifaximin	5	108.3	5.6	113.6	3.7	115.5	4.2	/	/	/	/	/
Rotenone	5	112.2	11.3	108.6	8.1	103.9	2.7	/	/	/	/	/

Saflufenacil	5	92.5	12.9	97.5	10.8	96.9	9.8	10	/	10	10	/
Sarafloxacin	5	90.9	13.1	111.6	14.8	106.9	1.9	/	/	/	/	/
Sparfloxacin	5	95.2	9.1	102.9	17.2	115.2	3.2	/	/	/	/	/
Sulfabenzamide	5	67.7	7.7	116.6	14.1	98.7	10.6	/	/	/	/	/
Sulfachinoxalin	5	79.8	10.5	77.6	5.2	82.1	5.5	/	/	/	10	/
Sulfadiazine	5	82.5	8.7	90.6	5.4	90.5	6.1	/	/	/	70	/
Sulfadoxine	5	101	8.5	111.2	11.5	105.8	5.2	/	/	/	60	/
Sulfamerazine	5	83.6	16.6	84.7	16.5	90	4.9	/	/	/	/	/
Sulfamethazine	5	75.4	12.2	102.2	16.1	109.5	10.4	/	/	/	25	/
Sulfamethoxazole	5	83.8	2.6	79.9	3.5	102.8	3.4	/	/	/	10	/
Sulfamethoxypyridazine	5	103.2	16.3	105.9	8.7	92.7	7.4	/	/	/	10	/
Sulfaphenazole	5	108.1	11.4	104.7	4.2	96.7	4.9	/	/	/	10	/
Sulfapyridine	5	77.7	10.1	110.2	4.5	90.8	7.3	/	/	/	10	/
Sulfentrazone	5	101.5	4.7	100.9	2.5	103.6	3.5	/	/	/	/	/
Sulfisoxazole	5	89.2	9.9	91.2	9.4	96.6	4.1	/	/	/	/	/
Tepraloxydim	5	87.3	10.9	113	18.7	98.9	10.9	/	/	/	/	/
Thiabendazole	5	101.4	15.8	103.9	4.2	94.7	5.9	/	/	/	100	50
Thiacloprid	5	104.8	1.9	106.1	14	102.3	5	50	/	/	50	30
Thidiazuron	5	89.3	10.3	103.3	13.2	94.8	3.7	/	/	/	20	50
Thiobencarb	5	111.4	9.3	112.2	5.4	102.8	3	/	10	/	/	50
Thiophanate	5	104.4	3.9	116.1	11.9	99.5	3.7	/	/	/	/	/
Thiophanate-methyl	5	107	7.1	118.7	8.1	104	5.9	/	50	/	/	/
Tolclofos-methyl	5	106.3	12.6	107.9	7.4	101	4.5	/	10	/	/	/
Tolfenpyrad	5	112.4	4.8	108.3	7.8	96.4	3.2	/	/	/	/	/
Triadimefon	5	102.9	3	90.3	4.3	105.7	3.7	10	/	/	50	/

Triasulfuron	5	66.1	11.9	79.6	14	97	5.2	/	/	/	20	20
Tricyclazole	5	69.4	5.3	78.3	5.5	77.9	6.7	/	10	/	/	/
Troleandomycin	5	80.2	9.5	110.7	17.7	84.4	5.8	/	/	/	/	/
Tulobuterol	5	100.3	5	99.2	2.5	100.8	3.3	/	/	/	/	/
Zoxamide	5	102.6	15.6	119	6.9	97.2	4.5	/	10	/	/	/
Compound	LOQ (μg /kg)	10 μg /kg		20 μg /kg		50 μg /kg						
		Recovery (%)	RSD (%)	Recovery (%)	RSD (%)	Recovery (%)	RSD (%)					
Acetamiprid	10	119.1	8.2	98.1	7.3	102.5	6.8	10	200	/	100	300
Amidosulfuron	10	113.2	15.9	104.1	6.8	106.5	8.9	/	70	/	/	/
Bromuconazol	10	75.2	9.5	117.6	7.9	115.9	2.7	/	10	/	/	/
Chlorfluazuron	10	103.8	7.3	93.8	3.8	98.4	9.2	/	/	/	30	/
Chlorsulfuron	10	108.1	9.5	94	4.3	96.5	10.5	/	/	/	/	/
Clopidol	10	94.3	6.8	99.1	5.8	105.4	4.1	/	/	/	/	/
Cyclosulfamuron	10	74.6	16.7	109.6	7.5	89.5	10.8	/	/	/	/	/
Cyhalofop	10	88.6	5.7	89.2	5.5	90.6	5.8	/	10	/	/	/
Ethopabate	10	69.4	6.8	79.7	5.5	80.6	5.8	/	/	/	10	/
Fenbuconazole	10	80.6	8.9	88.1	4.7	88.9	5	/	50	/	10	/
Fleroxacin	10	114.1	10.3	100.4	5.1	105.7	5.1	/	/	/	/	/
Flonicamid	10	106.4	5.9	110.3	9	114.7	10.5	/	150	/	200	50
Florasulam	10	97	11.9	99.1	7.1	94.8	4.9	/	10	/	/	/
Fluopicolide	10	80.6	5	89.4	7.1	91.6	4.9	20	20	/	20	/
Mesosulfuron-methyl	10	78.8	10.5	84.8	4.4	85.9	7.5	/	20	/	10	/
Mesotrione	10	103.1	12.4	112	6.8	106.3	4.9	/	/	/	/	/
Monolinuron	10	99.2	11.4	101.9	9.1	94.5	1.6	/	/	/	/	/
Moxidectin	10	106.3	6.8	110.5	4.2	110.8	5	40	/	/	40	/

Orthosulfamuron	10	73.6	11.6	102	16.1	101.6	2.9	/	/	/	/	/
Oxadiargyl	10	73.6	17.6	95.9	12.9	93.8	10.5	/	10	/	/	/
Prosulfuron	10	97.6	10.2	107.2	10.8	98.4	6.7	/	20	/	50	/
Rimsulfuron	10	103.8	12.8	102.2	8.9	105.7	5.7	/	20	/	/	/
Spiramycin	10	94.1	6.2	99.5	4.6	98.1	5.5	200	200	/	200	/
Sulcotrione	10	84.6	10.7	79.1	4.5	83.7	4.9	/	/	/	/	/
Sulfachloropyridazine	10	77.9	10.5	95.9	7.4	106.4	5.3	/	/	/	/	/
Sulfathiazole	10	97.6	12.6	110.1	6.5	93.8	5.1	/	/	/	90	/
Tolfenamic acid	10	85.8	10.4	102.5	10.4	91.8	4.3	/	50	/	50	/
Triallate	10	104.4	16.2	101.9	4.7	105.4	6.5	/	/	/	/	/
Tribenuron-methyl	10	103.5	11.4	89.6	6.2	84.9	3.8	/	10	10	10	/
Trifloxystrobin	10	63.5	8.5	70.6	8.8	69.5	5.9	/	/	/	/	/

Note: / means no MRLs value.
